# Adverse Events of Cannabidiol Use in Patients With Epilepsy: A Systematic Review and Meta-analysis

**DOI:** 10.1001/jamanetworkopen.2023.9126

**Published:** 2023-04-20

**Authors:** Asra Fazlollahi, Mahdi Zahmatyar, Mahta ZareDini, Behnam Golabi, Seyed Aria Nejadghaderi, Mark J. M. Sullman, Koroush Gharagozli, Ali-Asghar Kolahi, Saeid Safiri

**Affiliations:** 1 Student Research Committee, Tabriz University of Medical Sciences, Tabriz, Iran; 2 Research Center for Integrative Medicine in Aging, Aging Research Institute, Tabriz University of Medical Sciences, Tabriz, Iran; 3 Social Determinants of Health Research Center, Tabriz University of Medical Sciences, Tabriz, Iran; 4 Systematic Review and Meta-analysis Expert Group, Universal Scientific Education and Research Network, Tehran, Iran; 5 Department of Life and Health Sciences, University of Nicosia, Nicosia, Cyprus; 6 Department of Social Sciences, University of Nicosia, Nicosia, Cyprus; 7 Brain Mapping Research Center, Shahid Beheshti University of Medical Sciences, Tehran, Iran; 8 Social Determinants of Health Research Center, Shahid Beheshti University of Medical Sciences, Tehran, Iran; 9 Neurosciences Research Center, Aging Research Institute, Tabriz University of Medical Sciences, Tabriz, Iran; 10 Department of Community Medicine, Faculty of Medicine, Tabriz University of Medical Sciences, Tabriz, Iran

## Abstract

**Importance:**

Epilepsy is one of the most common neurologic disorders globally. Cannabidiol (CBD) has been approved for the treatment of epilepsy, but its use has been associated with several different adverse events (AEs).

**Objective:**

To investigate the frequency and risk of AEs developing in patients with epilepsy who are using CBD.

**Data Sources:**

PubMed, Scopus, Web of Science, and Google Scholar were searched for relevant studies published from database inception up to August 4, 2022. The search strategy included a combination of the following keywords: (*cannabidiol* OR *epidiolex*) AND (*epilepsy* OR *seizures*).

**Study Selection:**

The review included all randomized clinical trials that investigated at least 1 AE from the use of CBD in patients with epilepsy.

**Data Extraction and Synthesis:**

Basic information about each study was extracted. *I*^2^ statistics were calculated using *Q* statistics to assess the statistical heterogeneity among the included studies. A random-effects model was used in cases of substantial heterogeneity, and a fixed-effects model was used if the *I*^2^ statistic for the AEs was lower than 40%. This study was conducted according to the Preferred Reporting Items for Systematic Reviews and Meta-analyses (PRISMA) guideline.

**Main Outcomes and Measures:**

Frequency of each AE and risk of developing each AE in patients with epilepsy using CBD.

**Results:**

Nine studies were included. Overall incidences of 9.7% in the CBD group and 4.0% in the control group were found for any grade AEs. The overall risk ratios (RRs) for any grade and severe grade AEs were 1.12 (95% CI, 1.02-1.23) and 3.39 (95% CI, 1.42-8.09), respectively, for the CBD group compared with the control group. Compared with the control group, the CBD group had a greater risk for incidence of serious AEs (RR, 2.67; 95% CI, 1.83-3.88), AEs resulting in discontinuation (RR, 3.95; 95% CI, 1.86-8.37), and AEs resulting in dose reduction (RR, 9.87; 95% CI, 5.34-14.40). Because most of the included studies had some risk of bias (3 raised some concerns and 3 were at high risk of bias), these findings should be interpreted with some caution.

**Conclusions and Relevance:**

In this systematic review and meta-analysis of clinical trials, the use of CBD to treat patients with epilepsy was associated with an increased risk of several AEs. Additional studies are needed to determine the safe and effective CBD dosage for treating epilepsy.

## Key Points

**Question:**

What are the frequency and risk of adverse events (AEs) developing in patients with epilepsy who are using cannabidiol (CBD)?

**Findings:**

In this systematic review and meta-analysis, the frequency of any grade AEs in patients with epilepsy was more than 2 times higher for those using CBD than for those receiving placebo. The risks of any grade AEs, severe grade AEs, serious AEs, AEs leading to discontinuation, and AEs leading to dose reduction were significantly higher in patients receiving CBD than for those receiving placebo.

**Meaning:**

The treatment of patients with epilepsy using CBD was associated with the development of several types of AEs.

## Teaser

This systematic review and meta-analysis investigates the frequency and risk of developing adverse events from cannabidiol use in patients with epilepsy.

## Introduction

Epilepsy is one of the most common neurologic disorders globally, with a lifetime point prevalence of 7.6 per 1000 population and an annual incidence of 67 per 100 000 population.^1^ Although most cases can be treated or go into remission with age, in approximately one-third of cases the seizures continue despite pharmacotherapy, surgical, or dietary interventions.^2,3,4,5,6^ Therefore, it is important to find new alternatives for treating epilepsy.

Cannabidiol (CBD) is one of the naturally occurring compounds, known as cannabinoids, that are produced from the cannabis plant. In contrast to Δ8-tetrahydrocannabinol, CBD is not intoxicating at typical doses and lacks euphoric and other psychotropic effects.^7,8^ Cannabidiol is approved by both the US Food and Drug Administration and the European Medicines Agency as an additional therapy for severe forms of epilepsy, such as Dravet syndrome and Lennox-Gastaut syndrome.^9,10^

Previous studies have evaluated the efficacy and safety of CBD, or medicinal cannabis, in patients with epilepsy.^11,12^ Moreover, a study published in 2020 evaluated the adverse events (AEs) associated with CBD use across all medical indications,^13^ but to the best of our knowledge, no systematic review has focused on the AEs associated with CBD use in patients with epilepsy. Moreover, there is a need to update the previously published systematic reviews and to address their limitations. Therefore, we conducted a systematic review and meta-analysis to evaluate the AEs associated with CBD use in patients with epilepsy.

## Methods

### Search Strategy

For this systematic review and meta-analysis, PubMed, Scopus, and the Web of Science databases were searched for articles published from database inception to August 4, 2022, to identify publications reporting any AEs following treatment with CBD. In addition, the first 10 pages of the Google Scholar search engine were manually searched for grey literature. No filters were applied to any of the search fields, such as date, study type, or language. Backward and forward citation searches of all included studies were also performed, which means we screened all cited references of the included studies and all publications citing them to discover other qualified studies. In addition, studies included in similar previous systematic reviews were screened to identify whether there were any additional eligible articles. The searches were performed by 1 author (A.F.) and then double-checked by other authors (S.A.N. and S.S.). The search strategy included a combination of the following keywords: (*cannabidiol* OR *epidiolex*) AND (*epilepsy* OR *seizures*). A detailed description of the stages of the search for each database is given in eTable 1 in Supplement 1. The current study was approved by the ethics committee of the Shahid Beheshti University of Medical Sciences. This study was conducted according to the Preferred Reporting Items for Systematic Reviews and Meta-analyses (PRISMA) guideline.^14^

### Study Selection

All articles identified through the electronic and manual searches were exported to EndNote, version 20 (Clarivate), and any duplicates were removed. Two authors (A.F. and M. ZareDini) independently screened the title and abstract of the articles and excluded those that were irrelevant. In the next step, the same 2 authors reviewed the full texts of the remaining articles. Any discrepancies were resolved by discussion or consultation with other authors.

Studies were included if they were randomized clinical trials (RCTs) investigating at least 1 AE associated with CBD use in patients with epilepsy. All classifications of epilepsy were included, with no age restriction. Studies were excluded if they were not RCTs, did not consider the AEs of CBD, or included patients with diseases other than epilepsy.

### Data Extraction

Data extraction was conducted using previously designed Microsoft Office Excel forms (Microsoft Corp). Two reviewers (M. Zahmatyar and B.G.) independently extracted the following information from each included study: (1) the basic information about the study, including title, first author’s name, country, and publication date; (2) the characteristics of the participants, including study population, sample size, age, sex, type of epilepsy, and medications used for the treatment of epilepsy; and (3) the total number and severity of all-cause and treatment-related AEs observed in both the experimental and control groups as well as the total number of AEs resulting in discontinuation or dose reduction in both groups. Any disagreements were settled through discussion between the 2 reviewers or by conferring with a third reviewer (A.F.). Negative clinical events that developed in study participants after administration of CBD or placebo were considered to be AEs. We categorized the AEs according to the Common Terminology Criteria for Adverse Events, version 5.0.^15^

### Quality Assessment

Two reviewers (M. Zahmatyar and B.G.) independently appraised the risk of bias and the quality of the included articles using version 2 of the Cochrane risk-of-bias (RoB2) tool for randomized trials.^16^ The RoB2 tool classifies studies as having a high, low, or unclear risk of bias (some concerns) using the following 5 domains: randomization process, deviations from the intended interventions, missing outcome data, measurement of the outcome, and selection of the reported results. The overall risk-of-bias assessment in each study was also determined. Disagreements were resolved by discussion between the 2 reviewers (M. Zahmatyar and B.G.) or by consulting with a third reviewer (A.F.). We used the robvis package in R software, version 4.2.2 (R Foundation for Statistical Computing) to create the risk-of-bias graph.^17^

### Statistical Analysis

Stata, version 17.0 (StataCorp LLC) was used to perform the meta-analysis. We determined the frequency of mild, moderate, severe, and all grade AEs in both experimental and control groups using the metaprop command in Stata.^18^ The dichotomous raw data on the frequency of AEs in the intervention and control arms were extracted from each included study. We calculated the *I*^2^ statistics using *Q* statistics to assess the statistical heterogeneity among the included studies. The DerSimonian and Laird method was used for the random-effects models, and the inverse variance method was used for the fixed-effect models. A random-effects model was used in case of substantial heterogeneity, and a fixed-effects model was used if the *I*^2^ statistic was lower than 40% for the AEs.^19,20^ Continuity correction of 0.5 was used when the number of AEs for at least 1 arm was zero. Publication bias was only evaluated if at least 10 studies were included in the analysis.^21^ *P* < .05 was considered statistically significant.

## Results

### Study Selection

**Figure 1.**
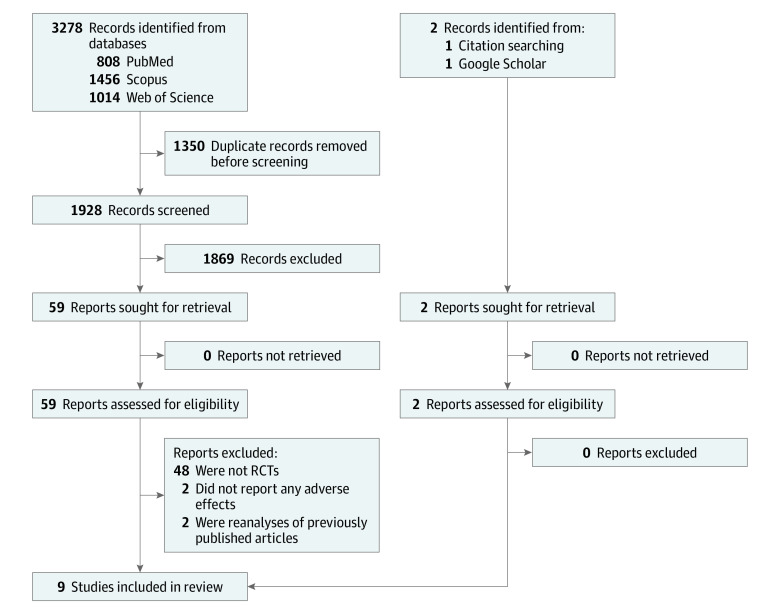
Study Selection Process RCT indicates randomized clinical trial.

The systematic search identified 3280 records. After the removal of 1350 duplicate records, the remaining 1930 publications were screened, and 61 studies were selected for full-text review (Figure 1). Following the evaluation of these studies for eligibility, 52 were excluded for the following reasons: 48 studies were not RCTs,^22,23,24,25,26,27,28,29,30,31,32,33,34,35,36,37,38,39,40,41,42,43,44,45,46,47,48,49,50,51,52,53,54,55,56,57,58,59,60,61,62,63,64,65,66,67,68,69^ 2 did not report AEs,^70,71^ and 2 were reanalyses of previously published articles.^72,73^ Finally, a total of 9 articles met the eligibility criteria and were included in the qualitative and quantitative synthesis.^74,75,76,77,78,79,80,81,82^

### Quality Assessment

**Figure 2.**
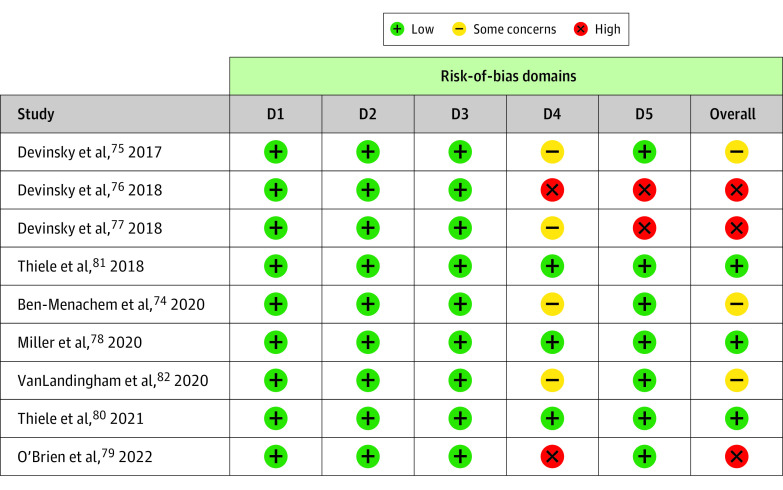
Summary of Risk-of-Bias Assessment for All Included Studies D indicates domain.

Bias due to randomization, deviation from the intended intervention, and missing data were low risk in all included trials. However, bias due to outcome measurement and the selection and reporting of results were high risk or there were some concerns in several studies. Overall, 3 trials had a low risk of bias,^78,80,81^ 3 had some concerns,^74,75,82^ and 3 had a high risk of bias^76,77,79^ (Figure 2; eTable 2 in Supplement 1).

### Study Characteristics

The included trials were published between 2017 and 2022 and involved patients with various forms of epilepsy (Dravet syndrome, Lennox-Gastaut syndrome, and tuberous sclerosis–associated epilepsy). One study^79^ used 390- and 195-mg transdermal CBD gels twice a day, whereas other studies^74,75,76,77,78,80,81,82^ used oral solutions twice daily. The daily oral dose of CBD ranged from 5 to 50 mg/kg, and the duration of treatment ranged from 3 to 16 weeks. Most of the studies were multicenter, and the age of participants ranged from 1.1 to 56.8 years (Table 1). The numbers of previous and concomitant antiepileptic drugs were fairly similar between the experimental and control arms (eTable 3 in Supplement 1). The concomitant antiepileptic drugs included valproate, clobazam, lamotrigine, levetiracetam, rufinamide, vigabatrin, stiripentol, lacosamide, ethosuximide, topiramate, zonisamide, oxcarbazepine, carbamazepine, lorazepam, clonazepam, eslicarbazepine, perampanel, and phenobarbital (eTable 4 in Supplement 1).

**Table 1.** Baseline Characteristics of the Studies Included in the Meta-analysis

Source	Study design (trial registration No.)	Study population	Type of CBD	Total daily CBD dose	Length of treatment	Safety follow-up duration	Sample size, No.	CBD, No.	Placebo, No.	Male, No. (%)	Female, No. (%)	Age, mean (SD), y
Ben-Menachem et al,^74^ 2020	Phase 2, 2-arm, parallel-group, double-blind, randomized, placebo-controlled DDI trial (NCT02607891)	Patients with epilepsy from Spain, the Netherlands, and Sweden, aged 16-55 y, already receiving a stable dose of stiripentol or valproate and having experienced at least 1 countable seizure of any type within the last 2 mo	Provided by GW Pharmaceuticals (100 mg/mL of oral solution)	20 mg/kg	24 d	NA	34	28	6	CBD: 17^62^; placebo: 5^84^	CBD: 11^40^; placebo: 1^18^	CBD: 30.1 (11.1); placebo: 26.9 (7.0)
Devinsky et al,^75^ 2017	Randomized, double-blind, placebo-controlled trial (NCT02091375)	Children and young adults from the US and Europe, aged 2-18 y, with poorly controlled Dravet syndrome	Provided by GW Pharmaceuticals (100 mg/mL of oral solution)	20 mg/kg	14 wk	4 wk	120	61	59	CBD: 35^58^; placebo: 27^47^	CBD: 26^44^; placebo: 32^55^	CBD: 9.8 (4.8); placebo: 9.7 (4.7)
Devinsky et al,^76^ 2018	Phase 3, randomized, double-blind, placebo-controlled trial (NCT02224560)	Patients with Lennox-Gastaut syndrome from the US, Spain, UK, and France, aged 2-55 y, with at least 2 types of generalized seizures, including drop seizures, for at least 6 mo	Provided by GW Pharmaceuticals (100 mg/mL of oral solution)	10, 20 mg/kg	14 wk	4 wk	225	Total: 149; CBD 10 mg/kg: 73; CBD 20 mg/kg: 76	76	CBD: 85^58^; placebo: 44^59^	CBD: 64^44^; placebo: 32^43^	CBD 10 mg/kg: 15.4 (9.5); CBD 20 mg/kg: 16.0 (10.8); placebo: 15.3 (9.3)
Devinsky et al,^77^ 2018	Randomized, double-blind, placebo-controlled, parallel-group trial (NCT02091206)	Patients with Dravet syndrome from the US and UK, aged 4-10 y, taking at least 1 AED	Provided by GW Pharmaceuticals (25 or 100 mg/mL of oral solution)	5, 10, 20 mg/kg	3 wk	4 wk	34	Total: 27; CBD 5 mg/kg: 10; CBD 10 mg/kg: 8; CBD 20 mg/kg: 9	7	CBD: 11^42^; placebo: 5^72^	CBD: 16^60^; placebo: 2^29^	CBD 5 mg/kg: 7.2 (1.9); CBD 10 mg/kg: 7.4 (2.1); CBD 20 mg/kg: 8.7 (1.8); placebo: 7.0 (0.9)
Miller et al,^78^ 2020	Double-blind, randomized, placebo-controlled, parallel-group trial (NCT02224703)	Patients with poorly controlled Dravet syndrome from the US, Spain, Poland, the Netherlands, Australia, and Israel, aged 2 to 18 y, taking at least 1 AED	Provided by GW Pharmaceuticals (100 mg/mL of oral solution)	10, 20 mg/kg	14 wk	4 wk	198	Total: 133; CBD 10 mg/kg: 66; CBD 20 mg/kg: 67	65	CBD: 63^48^; placebo: 31^49^	CBD: 70^54^; placebo: 34^53^	CBD 10 mg/kg: 9.2 (4.3); CBD 20 mg/kg: 9.3 (4.3); placebo: 9.6 (4.6)
O’Brien et al,^79^ 2022	Phase 2A, randomized, double-blind, placebo-controlled trial (ACTRN 12616000510448)	Patients with drug-resistant focal seizures for at least 2 y from Australia and New Zealand, aged 18 to 70 y, in generally good health, BMI ranging from 18 to 35, averaged at least 3 observable focal seizures per month with not more than 20 consecutive seizure-free days	Provided by Zynerba Pharmaceuticals (synthetic transdermal gel)	195, 390 mg (approximately 2.6, 5.3 mg/kg, respectively)	12 wk	NA	188	Total: 125; CBD 195 mg: 63; CBD 390 mg: 62	63	CBD: 58 (46.4); placebo: 27 (42.9)	CBD: 67 (53.6); placebo: 36 (57.1)	CBD 195 mg: 37.0 (12.6); CBD 390 mg: 40.4 (12.3); placebo: 40.3 (13.4)
Thiele et al,^80^ 2021	Phase 3, double-blind, parallel-group, randomized clinical trial (NCT02544763)	Patients with tuberous sclerosis complex–related medication-resistant epilepsy from the US, Poland, Australia, Spain, the Netherlands, and UK, aged 1-65 y, taking at least 1 antiepileptic medication	Provided by GW Pharmaceuticals (100 mg/mL of oral solution)	25, 50 mg/kg	16 wk	4 wk	224	Total: 148; CBD 25 mg/kg: 75; CBD 50 mg/kg: 73	76	CBD: 86^59^; placebo: 45^60^	CBD: 62^43^; placebo: 31^42^	NA
Thiele et al,^81^ 2018	Phase 3, randomized, double-blind, placebo-controlled trial (NCT02224690)	Patients from the US, the Netherlands, and Poland, aged 2-55 y, with a clinical diagnosis of Lennox-Gastaut syndrome, evidence of more than 1 type of generalized seizure, including drop seizures, for at least 6 mo	Provided by GW Pharmaceuticals (100 mg/mL of oral solution)	20 mg/kg	14 wk	4 wk	171	86	85	CBD: 45^53^; placebo: 43^52^	CBD: 41^49^; placebo: 42^50^	CBD: 15.5 (8.7); placebo: 15.3 (9.8)
VanLandingham et al,^82^ 2020	Phase 2, randomized, double-blind, placebo-controlled trial (NCT02565108)	Adult patients with poorly controlled epilepsy from the UK and Spain, already taking a stable dose of clobazam	Provided by GW Pharmaceuticals (100 mg/mL of oral solution)	20 mg/kg	32 d	NA	20	16	4	CBD: 8 (50.0); placebo: 2 (50.0)	CBD: 8 (50.0); placebo: 2 (50.0)	CBD: 36.6 (8.5); placebo: 37.6 (10.7)

Abbreviations: AED, antiepileptic drug; BMI, body mass index (calculated as weight in kilograms divided by height in meters squared); CBD, cannabidiol; DDI, drug-drug interaction; NA, not applicable.

### Meta-analysis Results

#### Frequency of AEs

The number and percentage of AEs reported in each included study can be found in eTable 5 in Supplement 1. In the intervention group, the most common AE of any grade was somnolence (22.0%), followed by a decreased appetite (19.5%) and pyrexia (15.3%). In the controls, upper respiratory tract infection (11.8%), diarrhea (10.9%), and pyrexia (10.2%) were the most common AEs. The overall percentage of any grade AEs was higher in the CBD group than in the control group (9.7% vs 4.0%) (eFigure 1 in Supplement 1). In the CBD arm, the overall percentages were 11.1% for mild AEs, 3.1% for moderate AEs, and 1.2% for severe AEs. In the control arm, the overall percentages were 6.4% for mild AEs, 1.3% for moderate AEs, and 0.7% for severe AEs (eFigure 2 in Supplement 1). The percentage of AEs that led to the discontinuation of the trial was higher in the CBD arm than in the controls (2.4% vs 0.7%) (eFigure 3 in Supplement 1).

#### Any Grade AEs and Mild, Moderate, and Severe Grade AEs

**Figure 3.**
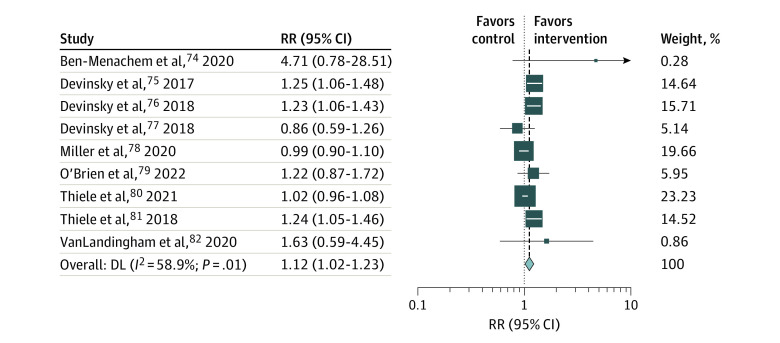
Risk Ratio (RR) for Any Grade Adverse Events for the Cannabidiol and Control Groups The studies by Devinsky et al^76,77^ and O’Brien et al^79^ have a high risk of bias; the studies by Devinsky et al,^75^ Ben-Menachem et al,^74^ and VanLandingham et al^82^ have some concerns; and the studies by Thiele et al^80,81^ and Miller et al^78^ have a low risk of bias. DL indicates DerSimonian and Laird.

The overall risk ratios (RRs) of any grade (from all 9 studies) and severe grade (in 5 studies^74,75,79,81,82^) AEs in the CBD group compared with the control group were 1.12 (95% CI, 1.02-1.23; *I*^2^ = 58.9%) for any grade and 3.39 (95% CI, 1.42-8.09; *I*^2^ = 3.5%) for severe grade (Figure 3; eFigure 4 in Supplement 1). For any grade AEs, the incidences of diarrhea (RR, 1.93; 95% CI, 1.44-2.58; *I*^2^ = 0.0%), somnolence (RR, 2.29; 95% CI, 1.61-3.25; *I*^2^ = 0.0%), decreased appetite (RR, 2.13; 95% CI, 1.48-3.06; *I*^2^ = 10.2%), and alanine transaminase (ALT) or aspartate aminotransferase (AST) elevation (12.29; 95% CI, 4.22-35.80; *I*^2^ = 0.0%) were significantly higher in the CBD group (Table 2; eFigures 5 and 6 in Supplement 1).

**Table 2.** Any Grade Adverse Events in the Meta-analysis^a^

Adverse event^b^	RR (95% CI)	*P* value
Infections and infestations		
Nasopharyngitis	1.02 (0.64-1.62)	.92
Pneumonia	2.15 (0.69-6.68)	.18
Upper respiratory tract infection	0.95 (0.60-1.49)	.81
Gastrointestinal disorders		
Diarrhea	1.93 (1.44-2.58)	<.001
Vomiting	1.22 (0.83-1.80)	.31
Nervous system disorders		
Somnolence	2.29 (1.61-3.25)	<.001
Status epilepticus	0.91 (0.49-1.67)	.75
General disorders		
Fatigue	1.36 (0.36-5.16)^c^	.65
Pyrexia	1.32 (0.93-1.86)	.11
Skin and subcutaneous tissue disorders		
Rash	3.02 (1.00-9.14)	.05
Metabolism and nutrition disorders		
Decreased appetite	2.13 (1.48-3.06)	<.001
Investigations		
ALT or AST elevation	12.29 (4.22-35.80)	<.001

Abbreviations: ALT, alanine aminotransferase; AST, aspartate aminotransferase; RR, risk ratio.

^a^ We categorized the adverse events according to the *Common Terminology Criteria for Adverse Events*.^15^

^b^ We included adverse events that were reported in 3 or more of the included studies.

^c^ The values from the random-effects model are reported.

Among mild grade AEs, the RR for the incidence of diarrhea was significantly higher in the CBD group (RR, 1.71; 95% CI, 1.21-2.42; *I*^2^ = 0.0%) (eFigure 7 in Supplement 1). Among moderate grade AEs, the risks of decreased appetite (RR, 3.25; 95% CI, 1.20-8.83; *I*^2^ = 0.0%) and somnolence (RR, 3.62; 95% CI, 1.45-9.04; *I*^2^ = 0.0%) were significantly higher in those receiving CBD (eFigure 8 in Supplement 1). There was no significant difference between the CBD group and controls in terms of risk of severe grade site-specific AEs (eFigure 9 and eTable 6 in Supplement 1).

#### Serious Grade AEs

Using data from 8 of the included studies,^74,75,76,77,78,79,80,81^ the overall RR for the incidence of serious AEs in the CBD group compared with the control group was 2.67 (95% CI, 1.83-3.88; *I*^2^ = 8.9%). Furthermore, in 2 studies, the CBD group had a higher RR for serious AEs (RR, 6.68; 95% CI, 1.63-27.38^80^ and RR, 4.94; 95% CI, 1.76-13.85)^81^ (eFigure 10 in Supplement 1).

#### AEs Resulting in Discontinuation or Dose Reduction

The incidence of AEs leading to the discontinuation of treatment in 8 studies^74,75,76,77,79,80,81,82^ were significantly higher in the CBD group than in the control group (RR, 3.95; 95% CI, 1.86-8.37; *I*^2^ = 0.0%) (eFigure 11 in Supplement 1). However, there were no significant differences between the CBD and control groups for AST or ALT elevation, diarrhea, and rashes leading to discontinuation of the trial (eFigure 12 in Supplement 1). The incidence of AEs that resulted in dose reduction in the 3 included studies^75,81,82^ was significantly higher in the CBD group than in the control group (RR, 9.87; 95% CI, 5.34-14.40; *I*^2^ = 0.0%) (eFigure 13 in Supplement 1).

#### Subgroup Analysis

A subgroup analysis was performed by quality of the included studies. The results showed that the pooled RRs for incidence of overall any grade AEs were 1.05 (95% CI, 0.96-1.16; *I*^2^ = 62.9%) in studies with low risk of bias, 1.15 (95% CI, 0.95-1.39; *I*^2^ = 33.6%) in studies with high risk of bias, and 1.35 (95% CI, 0.95-1.92; *I*^2^ = 12.8%) in studies with some concerns (eFigure 14 in Supplement 1).

## Discussion

The current study showed that the frequency of any grade AEs in patients with epilepsy was more than 2 times higher for those receiving CBD than for the controls, with a notably increased risk of ALT or AST elevation, decreased appetite, diarrhea, and somnolence in those receiving CBD. The risks of any grade AEs, severe grade AEs, serious AEs, AEs leading to the discontinuation of the trial, and AEs leading to dose reduction were significantly higher in patients receiving CBD than in controls.

The current study also indicated that for those receiving CBD, the overall percentage of any grade AEs was 9.7%, severe grade AEs was 1.2%, and AEs leading to the discontinuation of the trial was 2.4%. A previous systematic review and meta-analysis^11^ of RCTs involving patients with uncontrolled epilepsy showed that the frequency of any grade AEs was 55.7% and the frequency of serious AEs was 17.6%. The lower percentage of AE frequency found in our study may be as a result of including more studies in our analysis (9 vs 4).^11^ In addition, differences in the inclusion criteria in the systematic reviews and definitions of AEs can lead to variations in the AE incidence. Moreover, a systematic review by Bilbao and Spanagel^83^ on the safety and efficacy of cannabinoids for different diseases showed that serious or severe AEs occurred in 4.5% of those receiving CBD, which was lower than for other cannabinoids, such as dronabinol (5.4%) and nabilone (6.3%). Therefore, it seems that CBD is a relatively safe option compared with other cannabinoids.

The current study showed that use of CBD was associated with a 1.2 times increase in the incidence of any grade AEs, 3.39 times increase in the incidence of severe grade AEs, 2.67 times increase in the incidence of serious AEs, 3.95 times increase in the incidence of AEs leading to discontinuation of the trial, and 9.87 times increase in the incidence of AEs resulting in dose reduction compared with controls. Similarly, a systematic review by Lattanzi et al^11^ found an RR of 1.22 (95% CI, 1.11-1.33) for the incidence of AEs in those receiving CBD compared with controls. Moreover, another meta-analysis^84^ of 3 trials on the safety of adjunctive CBD in patients with Dravet syndrome showed that adding CBD was nonsignificantly associated with an increased risk of developing any type of AE (RR, 1.06; 95% CI, 0.87-1.28). Their nonsignificant result may be because they only included Dravet syndrome, had a low sample size, and included articles in the analysis that evaluated the effects of CBD that was administered in combination with other drugs.^84^ Another meta-analysis^85^ of 2 trials with 396 participants showed a significantly higher RR for AE development in patients with Lennox-Gastaut syndrome being treated with CBD (RR, 1.24; 95% CI, 1.11-1.38). Furthermore, the results of a systematic review and meta-analysis^86^ on the efficacy and safety of CBD for pediatric refractory epilepsy found an increased risk of overall AEs of 1.81 (95% CI, 1.33-2.46) and of serious AEs of 2.86 (95% CI, 1.63-5.05). The risk of any grade and serious AEs in our study were lower than the above-mentioned study (1.12 vs 1.81 for any grade AEs and 2.67 vs 2.86 for serious AEs),^86^ which may be because of the inclusion of different populations and AE definitions.

The current study found that there was a significantly increased incidence of diarrhea, somnolence, decreased appetite, and ALT or AST elevation among those receiving CBD. Similarly, 3 previous systematic reviews and meta-analyses^11,84,85^ of patients with uncontrolled epilepsy (ie, Dravet syndrome and Lennox-Gastaut syndrome) showed that CBD was significantly associated with increased risks of somnolence, decreased appetite, diarrhea, and increased serum aminotransferases. Although Ben-Menachem et al^74^ did not show notable increases in the RRs for ALT or AST elevation in their study, in most trials, CBD was associated with an increased RR of ALT or AST elevation when compared with the placebo group. These differences may be due to the fact that the studies were examining the effect of CBD treatment after a different period (25 days vs 14 weeks) in low numbers of patients with wide age ranges.^75,80,81^ Thiele et al^80^ found a significant RR for elevated ALT or AST levels as a result of treatment with high-dose CBD (25 mg/kg daily and 50 mg/kg daily) using a long treatment period compared with other studies (16 vs 14 weeks).

Among mild, moderate, and severe manifestations of somnolence, the moderate type was statistically significantly higher than in the control group in analyses of the pooled data from 4 studies^75,78,80,81^ (RR, 3.62; 95% CI, 1.45-9.04). In most of these studies, taking clobazam was associated with somnolence.^75,78,80,81^ For instance, in 1 of the RCTs,^75^ approximately 80% of the patients with reported somnolence were also taking clobazam. In another study,^80^ 41% of the CBD group taking clobazam concomitantly reported somnolence as an AE in contrast to 12.5% among those not taking clobazam. Drug-drug interactions between clobazam and CBD were associated with an almost 3-fold increase in exposure to clobazam’s active metabolite, N-desmethyl clobazam, and an approximately 1.5-fold increase in CBD’s active metabolite, 7-hydroxy cannabidiol, in healthy volunteers. However, several studies involving patients with epilepsy did not demonstrate any drug-drug interaction between CBD and clobazam, whereas exposure to N-desmethyl clobazam was enhanced by 2- to 3-fold, probably resulting from the inhibition of CYP2C19 by CBD, which leads to clobazam dose adjustments in the presence of CBD.^78,81,82,87^

A previous systematic review and meta-analysis of RCTs,^13^ which used the RoB1 tool for quality assessment, showed that in most domains, most studies had low risks of bias, and only 2 studies had high risks of bias in the selective outcome reporting domains. Similarly, in our study, which used the RoB2 tool, we found that there were high risks of bias in the selection of reported results and in the measurement of outcomes, whereas other domains had low risks of bias. The study by Talwar et al^86^ also showed that the 6 included RCTs all had a low risk of bias. Overall, it is suggested that future studies carefully consider the measurement of outcomes, register the RCT protocol, and report both significant and nonsignificant outcomes.

### Limitations

Our study has some limitations, which should be considered when interpreting the results. Although most of the participants were treatment-resistant patients with epilepsy, there was substantial heterogeneity in the study population in terms of age, severity of the disease, CBD dosage, source of CBD, and even its route of administration. The use of antiepileptic drugs, other than CBD, and the use of different dosages can influence the AEs that develop following the use of CBD. In addition, we only included AEs in the meta-analysis that were reported in at least 3 studies, so AEs that were only reported in 1 or 2 of the included studies were not reported. Furthermore, small-study bias and publication bias were not evaluated because fewer than 10 studies were included.^21^ We could not investigate the association between CBD plasma levels and AEs, but this should be investigated in future RCTs and meta-analyses. In addition, we did not limit the selection criteria to only major RCTs, which might lead to the inclusion of studies in different phases, such as phase 2 studies with small numbers of patients and a short observation period. Furthermore, we did not perform a subgroup analysis by type of epilepsy, dose, or duration of the treatment because of the limited number of included studies and/or several studies not providing the relevant information.

## Conclusions

In this systematic review and meta-analysis, the use of CBD to treat patients with epilepsy was associated with the development of several AEs, such as somnolence, diarrhea, decreased appetite, and AST or ALT elevation. Future research needs to investigate the therapeutic effects of CBD and AEs in the presence of various dosages of other antiepileptic drugs in order to achieve a safe and effective dose for treatment-resistant patients with epilepsy.

## Notes

Supplement 1. **eTable 1.** Search Strategy Used With the Four Databases **eTable 2.** Quality Assessment of the Included Studies **eTable 3.** Baseline Characteristics of the Studies Included in the Meta-analysis **eTable 4.** Concomitant Anti-epileptic Drugs Taken by Participants in the Included Trials, N (%) **eTable 5.** Adverse Events Observed in the Included Trials, N (%) **eTable 6.** Graded Adverse Events in the Meta-analysis **eFigure 1.** Percentages of Any-Grade Adverse Events for the Cannabidiol and Control Groups **eFigure 2.** Percentages of Mild, Moderate and Severe Adverse Events for the Cannabidiol and Control Groups **eFigure 3.** Percentages of Adverse Events Leading to the Discontinuation of the Trial for the Cannabidiol and Control Groups **eFigure 4.** Forest Plot of the Risk Ratios for Severe Grade Adverse Events for the Cannabidiol and Control Groups **eFigure 5.** Forest Plots of the Risk Ratios for Any-Grade Adverse Events, Including ALT or AST Elevation, Decreased Appetite, Diarrhea, Fatigue, Nasopharyngitis, and Pneumonia for the Cannabidiol and Control Groups **eFigure 6.** Forest Plots of the Risk Ratios for Any-Grade Adverse Events, Including Pyrexia, Rash, Somnolence, Status Epilepticus, Upper Respiratory Tract Infection, and Vomiting for the Cannabidiol and Control Groups **eFigure 7.** Forest Plot of the Risk Ratios for Mild Adverse Events, Including Decreased Appetite, Diarrhea, Nasopharyngitis, Pyrexia, Somnolence, and Vomiting for the Cannabidiol and Control Groups **eFigure 8.** Forest Plot of the Risk Ratios for Moderate Adverse Events, Including Decreased Appetite, Diarrhea, Pyrexia, Somnolence, and Vomiting for the Cannabidiol and Control Groups **eFigure 9.** Forest Plot of the Risk Ratios for Severe Adverse Events, Including Decreased Appetite, Diarrhea, and Somnolence for the Cannabidiol and Control Groups **eFigure 10.** Forest Plot of the Risk Ratio for Serious Adverse Events for the Cannabidiol and Control Groups **eFigure 11.** Forest Plot of the Risk Ratio for Adverse Events Leading to the Discontinuation the Trial for the Cannabidiol and Control Groups **eFigure 12.** Forest Plot of the Risk Ratios for Adverse Events Leading to the Discontinuation of the Trial, Including ALT or AST Elevation, Diarrhea, and Rash for the Cannabidiol and Control Groups **eFigure 13.** Forest Plot of the Risk Ratios for Adverse Events Leading to Dose Reduction for the Cannabidiol and Control Groups **eFigure 14.** Forest Plot of the Risk Ratio for Any-Grade Adverse Events for the Cannabidiol and Control Groups by Quality of the Included Studies

Supplement 2.
Data Sharing Statement
