# Supplementary material for: Adverse Events of Cannabidiol Use in Patients With Epilepsy: A Systematic Review and Meta-analysis
Source: JAMA Netw Open. 2023 Apr 20;6(4):e239126. doi: 10.1001/jamanetworkopen.2023.9126 (PMC10119734; doi:10.1001/jamanetworkopen.2023.9126)
Supplement: Supplement 1. — eTable 1. Search Strategy Used With the Four Databases eTable 2. Quality Assessment of the Included Studies eTable 3. Baseline Characteristics of the Studies Included in the Meta-analysis eTable 4. Concomitant Anti-epileptic Drugs Taken by Participants in the Included Trials, N (%) eTable 5. Adverse Events Observed in the Included Trials, N (%) eTable 6. Graded Adverse Events in the Meta-analysis eFigure 1. Percentages of Any-Grade Adverse Events for the Cannabidiol and Control Groups eFigure 2. Percentages of Mild, Moderate and Severe Adverse Events for the Cannabidiol and Control Groups eFigure 3. Percentages of Adverse Events Leading to the Discontinuation of the Trial for the Cannabidiol and Control Groups eFigure 4. Forest Plot of the Risk Ratios for Severe Grade Adverse Events for the Cannabidiol and Control Groups eFigure 5. Forest Plots of the Risk Ratios for Any-Grade Adverse Events, Including ALT or AST Elevation, Decreased Appetite, Diarrhea, Fatigue, Nasopharyngitis, and Pneumonia for the Cannabidiol and Control Groups eFigure 6. Forest Plots of the Risk Ratios for Any-Grade Adverse Events, Including Pyrexia, Rash, Somnolence, Status Epilepticus, Upper Respiratory Tract Infection, and Vomiting for the Cannabidiol and Control Groups eFigure 7. Forest Plot of the Risk Ratios for Mild Adverse Events, Including Decreased Appetite, Diarrhea, Nasopharyngitis, Pyrexia, Somnolence, and Vomiting for the Cannabidiol and Control Groups eFigure 8. Forest Plot of the Risk Ratios for Moderate Adverse Events, Including Decreased Appetite, Diarrhea, Pyrexia, Somnolence, and Vomiting for the Cannabidiol and Control Groups eFigure 9. Forest Plot of the Risk Ratios for Severe Adverse Events, Including Decreased Appetite, Diarrhea, and Somnolence for the Cannabidiol and Control Groups eFigure 10. Forest Plot of the Risk Ratio for Serious Adverse Events for the Cannabidiol and Control Groups eFigure 11. Forest Plot of the Risk Ratio for Adverse Events Leading to the Disconti [file jamanetwopen-e239126-s001.pdf]

Supplementary Online Content

Fazlollahi A, Zahmatyar M, ZareDini M, et al. Adverse events of cannabidiol in patients with epilepsy: a systematic review and meta-analysis. *JAMA Netw Open*. 2023;6(4):e239126. doi:10.1001/jamanetworkopen.2023.9126

eTable 1. Search Strategy Used With the Four Databases

eTable 2. Quality Assessment of the Included Studies

eTable 3. Baseline Characteristics of the Studies Included in the Meta-analysis

eTable 4. Concomitant Anti-epileptic Drugs Taken by Participants in the Included Trials, N (%)

eTable 5. Adverse Events Observed in the Included Trials, N (%)

eTable 6. Graded Adverse Events in the Meta-analysis

eFigure 1. Percentages of Any-Grade Adverse Events for the Cannabidiol and Control Groups

eFigure 2. Percentages of Mild, Moderate and Severe Adverse Events for the Cannabidiol and Control Groups

eFigure 3. Percentages of Adverse Events Leading to the Discontinuation of the Trial for the Cannabidiol and Control Groups

eFigure 4. Forest Plot of the Risk Ratios for Severe Grade Adverse Events for the Cannabidiol and Control Groups

eFigure 5. Forest Plots of the Risk Ratios for Any-Grade Adverse Events, Including ALT or AST Elevation, Decreased Appetite, Diarrhea, Fatigue, Nasopharyngitis, and Pneumonia for the Cannabidiol and Control Groups

eFigure 6. Forest Plots of the Risk Ratios for Any-Grade Adverse Events, Including Pyrexia, Rash, Somnolence, Status Epilepticus, Upper Respiratory Tract Infection, and Vomiting for the Cannabidiol and Control Groups

eFigure 7. Forest Plot of the Risk Ratios for Mild Adverse Events, Including Decreased Appetite, Diarrhea, Nasopharyngitis, Pyrexia, Somnolence, and Vomiting for the Cannabidiol and Control Groups

eFigure 8. Forest Plot of the Risk Ratios for Moderate Adverse Events, Including Decreased Appetite, Diarrhea, Pyrexia, Somnolence, and Vomiting for the Cannabidiol and Control Groups

eFigure 9. Forest Plot of the Risk Ratios for Severe Adverse Events, Including Decreased Appetite, Diarrhea, and Somnolence for the Cannabidiol and Control Groups

eFigure 10. Forest Plot of the Risk Ratio for Serious Adverse Events for the Cannabidiol and Control Groups

eFigure 11. Forest Plot of the Risk Ratio for Adverse Events Leading to the Discontinuation the Trial for the Cannabidiol and Control Groups

eFigure 12. Forest Plot of the Risk Ratios for Adverse Events Leading to the Discontinuation of the Trial, Including ALT or AST Elevation, Diarrhea, and Rash for the Cannabidiol and Control Groups

eFigure 13. Forest Plot of the Risk Ratios for Adverse Events Leading to Dose Reduction for the Cannabidiol and Control Groups

eFigure 14. Forest Plot of the Risk Ratio for Any-Grade Adverse Events for the Cannabidiol and Control Groups by Quality of the Included Studies

eReferences

This supplementary material has been provided by the authors to give readers additional information about their work.

© 2023 Fazlollahi A et al. *JAMA Network Open.*

eTable 1. Search strategy used with the four databases.

Database (Search date)	Step	Search Strategy	Number of Results
PubMed (8.4.2022)	#1	“cannabidiol”[mh] OR “cannabidiol”[tiab] OR “CBD”[tiab] OR “epidiolex”[tiab] OR “epidyolex”[tiab]	11,078
	#2	“epilepsy”[mh] OR “epileps*”[tiab] OR “epileptic” OR “seizures”[mh] OR “seizur*”[tiab]	252,693
	#3	#1 AND #2	808
Scopus (8.4.2022)	#1	TITLE-ABS-KEY (“cannabidiol” OR “CBD” OR “epidiolex” OR “epidyolex”)	21,744
	#2	TITLE-ABS-KEY (“epileps*” OR “eplileptic” OR “seizur*”)	361,580
	#3	#1 AND #2	1,456
Web of Science (8.4.2022)	#1	TS=(“cannabidiol” OR “CBD” OR “epidiolex” OR “epidyolex”)	16,676
	#2	TS=(“epileps*” OR “epileptic” OR “seizur*”)	247,805
	#3	#1 AND #2	1,014
Google Scholar (8.18.2022)		((“cannabidiol” OR “CBD” OR “epidiolex” OR “epidyolex”) AND (“epilepsy” OR “epilepsies” OR “espilepsia” OR “epileptic” OR “seizure” OR “seizures”))	27,600

© 2023 Fazlollahi A et al. *JAMA Network Open.*

eTable 2. Quality assessment of the included studies.

Study ID	D1	D2	D3	D4	D5	Overall bias
Devinsky et al. 2017 (1)	Low	Low	Low	Some concerns	Low	Some concerns
Devinsky et al. 17, 2018 (2)	Low	Low	Low	High	High	High
Devinsky et al. 14, 2018 (3)	Low	Low	Low	Some concerns	High	High
Thiele et al. 2018 (4)	Low	Low	Low	Low	Low	Low
Ben-Menachem et al. 2020 (5)	Low	Low	Low	Some concerns	Low	Some concerns
Miller et al. 2020 (6)	Low	Low	Low	Low	Low	Low
VanLandingham et al. 2020 (7)	Low	Low	Low	Some concerns	Low	Some concerns
Thiele et al. 2020 (8)	Low	Low	Low	Low	Low	Low
O'Brien et al. 2022 (9)	Low	Low	Low	High	Low	High

D1: Bias arising from the randomization process

D2: Bias due to deviations from intended interventions

D3: Bias due to missing outcome data

D4: Bias in measurement of the outcome

D5: Bias in selection of the reported results

RoB2 overall risk of bias judgment

Low risk of bias → The study is judged to be at low risk of bias for all domains for this result.

Some concerns → The study is judged to raise some concerns in at least one domain for this result, but not to be at high risk of bias for any domain.

High risk of bias → The study is judged to be at high risk of bias in at least one domain, or to have some concerns for multiple domains in a way that substantially lowers confidence in the result.

© 2023 Fazlollahi A et al. *JAMA Network Open.*

eTable 3. Baseline characteristics of the studies included in the meta-analysis.

Study ID	BMI (kg/m2)	No. of previous antiepileptic drugs	No. of concomitant antiepileptic drugs
Ben-Menachem et al. 2020 (5)	CBD: 26.8±5.4<br>Placebo: 27.0±5.3	N/A	N/A
Devinsky et al. 2017 (1)	CBD: 18.3±4.5<br>Placebo: 19.1±4.7	CBD: 4.6±4.3<br>Placebo: 4.6±3.3	CBD: 3.0±1.0<br>Placebo: 2.9±1.0
Devinsky et al. 17, 2018 (2)	N/A	CBD10: 6 (0–21)<br>CBD20: 6 (1–18)<br>Placebo: 6 (1–22)	CBD10: 3 (1–5)<br>CBD20: 3 (0–5)<br>Placebo: 3 (1–5)
Devinsky et al. 14, 2018 (3)	CBD5: 18.9±4.4<br>CBD10: 16.1±1.5<br>CBD20: 16.1±2.3<br>Placebo: 18.7±4.0	N/A	CBD5: 2.6±1.1<br>CBD10: 2.8±0.5<br>CBD20: 2.8±0.8<br>Placebo: 2.1±0.9
Miller et al. 2020 (6)	N/A	CBD10: 4 (0-19)<br>CBD20: 4 (0-11)<br>Placebo: 4 (0-11)	CBD10: 3 (1-5)<br>CBD20: 3 (1-4)<br>Placebo: 3 (1-5)
O’Brien et al. 2020 (9)	CBD195: 26.03±4.59<br>CBD390: 25.77±4.57<br>Placebo: 25.97±4.54	N/A	N/A
Thiele et al. 2020 (8)	N/A	CBD25: 4 (0-13)<br>CBD50: 4 (0-13)<br>Placebo: 4 (0-15)	CBD25: 3 (0-4)<br>CBD50: 3 (1-5)<br>Placebo: 3 (1-5)
Thiele et al. 2018 (4)	N/A	CBD: 6 (1–18)<br>Placebo: 6 (0–28)	CBD: 3 (1–5)<br>Placebo: 3 (1–4)
Vanlandingham et al. 2020 (7)	CBD: 28.25±5.2<br>Placebo: 25.98±5.8	N/A	N/A

Abbreviations: BMI: body mass index, CBD: cannabidiol; N/A: not applicable

eTable 4. Concomitant anti-epileptic drugs taken by participants in the included trials, n (%).

**Study ID**	Ben-Menachem et al. 2020 (5)	Devinsky et al. 2017 (1)	Devinsky et al. 17, 2018 (2)	Devinsky et al. 14, 2018 (3)	Miller et al. 2020 (6)	O'Brien et al. 2022 (9)	Thiele et al. 2020 (8)	Thiele et al. 2018 (4)	VanLandingham et al. 2020 (7)
**Valproate**	CBD: 21 (75)<br>Placebo: 4 (67)	CBD: 37 (61)<br>Placebo: 34 (58)	CBD10: 27 (37)<br>CBD20: 28 (37)<br>Placebo: 30 (39)	CBD5: 7 (70)<br>CBD10: 5 (63)<br>CBD20: 8 (89)<br>Placebo: 2 (29)	CBD10: 44 (67)<br>CBD20: 47 (70)<br>Placebo: 48 (74)	N/A	CBD25: 29 (39)<br>CBD50: 36 (49)<br>Placebo: 35 (46)	CBD: 36 (42)<br>Placebo: 33 (39)	CBD: 3 (18.8)<br>Placebo: 0
**Clobazam**	CBD: 10 (36)<br>Placebo: 0	CBD: 40 (66)<br>Placebo: 38 (64)	CBD10: 37 (51)<br>CBD20: 36 (47)<br>Placebo: 37 (49)	CBD5: 6 (60)<br>CBD10: 6 (75)<br>CBD20: 6 (67)<br>Placebo: 5 (71)	CBD10: 45 (68)<br>CBD20: 40 (60)<br>Placebo: 41 (63)	N/A	CBD25: 17 (23)<br>CBD50: 19 (26)<br>Placebo: 25 (33)	CBD: 41 (48)<br>Placebo: 43 (51)	CBD: 16 (100)<br>Placebo: 4 (100)
**Lamotrigine**	CBD: 6 (21)<br>Placebo: 2 (33)	N/A	CBD10: 22 (30)<br>CBD20: 20 (26)<br>Placebo: 25 (33)	N/A	N/A	N/A	N/A	CBD: 33 (38)<br>Placebo: 31 (36)	CBD: 4 (25.0)<br>Placebo: 1 (25.0)
**Levetiracetam**	CBD: 2 (7)<br>Placebo: 1 (17)	CBD: 16 (26)<br>Placebo: 17 (29)	CBD10: 22 (30)<br>CBD20: 24 (32)<br>Placebo: 23 (30)	CBD5: 3 (30)<br>CBD10: 3 (38)<br>CBD20: 3 (33)<br>Placebo: 1 (14)	CBD10: 19 (29)<br>CBD20: 21 (31)<br>Placebo: 14 (22)	N/A	CBD25: 19 (25)<br>CBD50: 22 (30)<br>Placebo: 24 (32)	CBD: 24 (28)<br>Placebo: 34 (40)	CBD: 7 (43.8)<br>Placebo: 2 (50.0)
**Rufinamide**	CBD: 2 (7)<br>Placebo: 2 (33)	N/A	CBD10: 19 (26)<br>CBD20: 26 (34)<br>Placebo: 20 (26)	N/A	N/A	N/A	N/A	CBD: 24 (28)<br>Placebo: 22 (26)	N/A
**Vigabatrin**	N/A	N/A	N/A	N/A	N/A	N/A	CBD25: 28 (37)<br>CBD50: 29 (40)<br>Placebo: 17 (22)	N/A	N/A
**Stiripentol**	CBD: 12 (43)<br>Placebo: 2 (33)	CBD: 30 (49)<br>Placebo: 21 (36)	N/A	CBD5: 1 (10)<br>CBD10: 2 (25)<br>CBD20: 2 (22)<br>Placebo: 2 (29)	CBD10: 25 (38)<br>CBD20: 22 (33)<br>Placebo: 24 (37)	N/A	N/A	N/A	N/A
**Study ID**	Ben-Menachem	Devinsky et al. 2017 (1)	Devinsky et al. 17, 2018 (2)	Devinsky et al. 14, 2018 (3)	Miller et al. 2020 (6)	O'Brien et al. 2022 (9)	Thiele et al. 2020 (8)	Thiele et al. 2018 (4)	VanLandingham et al. 2020 (7)

© 2023 Fazlollahi A et al. *JAMA Network Open.*

	et al. 2020 (5)								
**Lacosamide**	CBD: 6 (21) Placebo: 2 (33)	N/A	N/A	N/A	N/A	N/A	N/A	N/A	CBD: 3 (18.8) Placebo: 2 (50.0)
**Ethosuximide**	CBD: 2 (7) Placebo: 0	N/A	N/A	N/A	N/A	N/A	N/A	N/A	N/A
**Topiramate**	CBD: 2 (7) Placebo: 0	CBD: 16 (26) Placebo: 15 (25)	N/A	CBD5: 3 (30) CBD10: 3 (38) CBD20: 2 (22) Placebo: 2 (29)	CBD10: 11 (17) CBD20: 18 (27) Placebo: 17 (26)	N/A	N/A	N/A	N/A
**Zonisamide**	CBD: 2 (7) Placebo: 0	N/A	N/A	N/A	N/A	N/A	N/A	N/A	N/A
**Oxcarbazepine**	CBD: 1 (4) Placebo: 1 (17)	N/A	N/A	N/A	N/A	N/A	N/A	N/A	CBD: 2 (12.5) Placebo: 1 (25.0)
**Carbamazepine**	CBD: 1 (4) Placebo: 1 (17)	N/A	N/A	N/A	N/A	N/A	N/A	N/A	CBD: 4 (25.0) Placebo: 1 (25.0)
**Lorazepam**	CBD: 1 (4) Placebo: 1 (17)	N/A	N/A	N/A	N/A	N/A	N/A	N/A	N/A
**Clonazepam**	CBD: 2 (7) Placebo: 1 (17)	N/A	N/A	N/A	N/A	N/A	N/A	N/A	N/A
**Eslicarbazepine**	N/A	N/A	N/A	N/A	N/A	N/A	N/A	N/A	CBD: 3 (18.8) Placebo: 0
**Perampanel**	N/A	N/A	N/A	N/A	N/A	N/A	N/A	N/A	CBD: 0 Placebo: 1 (25.0)
**Phenobarbital**	N/A	N/A	N/A	N/A	N/A	N/A	N/A	N/A	CBD: 1 (6.3) Placebo: 0

Abbreviation: CBD: cannabidiol; N/A: not applicable.

© 2023 Fazlollahi A et al. *JAMA Network Open.*

eTable 5. Adverse events observed in the included trials, n (%).

**Study ID**	Ben-Menachem et al. 2020 (5)	Devinsky et al. 2017 (1)	Devinsky et al. 17, 2018 (2)	Devinsky et al. 14, 2018 (3)	Miller et al. 2020 (6)	O'Brien et al. 2022 (9)	Thiele et al. 2020 (8)	Thiele et al. 2018 (4)	VanLandingham et al. 2020 (7)
**Pneumonia**	N/A	N/A	N/A	CBD5: 0<br>CBD10: 1 (13)<br>CBD20: 1 (11)<br>Placebo: 0	CBD10: 6 (9)<br>CBD20: 4 (6)<br>Placebo: 2 (3)	N/A	CBD25: 2 (3)<br>CBD50: 2 (3)<br>Placebo: 1 (1)	N/A	N/A
**Upper respiratory tract infection**	N/A	CBD: 7 (11)<br>Placebo: 5 (8)	CBD10: 11 (16)<br>CBD20: 11 (13)<br>Placebo: 11 (14)	N/A	N/A	N/A	CBD25: 7 (9)<br>CBD50: 7 (10)<br>Placebo: 10 (13)	N/A	N/A
**Diarrhea**	CBD: 16 (57)<br>Placebo: 0	CBD: 19 (31)<br>Placebo: 6 (10)	CBD10: 7 (10)<br>CBD20: 12 (15)<br>Placebo: 6 (8)	N/A	CBD10: 11 (17)<br>CBD20: 18 (26)<br>Placebo: 8 (12)	CBD: 3 (2.4)	CBD25: 23 (31)<br>CBD50: 41 (56)<br>Placebo: 19 (25)	CBD: 16 (19)<br>Placebo: 7 (8)	CBD: 6 (37.5)<br>Placebo: 1 (25.0)
**Somnolence**	N/A	CBD: 22 (36)<br>Placebo: 6 (10)	CBD10: 14 (21)<br>CBD20: 25 (30)<br>Placebo: 4 (5)	CBD5: 2 (20)<br>CBD10: 3 (38)<br>CBD20: 0<br>Placebo: 1 (14)	CBD10: 16 (25)<br>CBD20: 16 (23)<br>Placebo: 9 (14)	N/A	CBD25: 10 (13)<br>CBD50: 19 (26)<br>Placebo: 7 (9)	CBD: 13 (15)<br>Placebo: 8 (9)	CBD: 2 (12.5)<br>Placebo: 0
**Pyrexia**	N/A	CBD: 9 (15)<br>Placebo: 5 (8)	CBD10: 6 (9)<br>CBD20: 10 (12)<br>Placebo: 12 (16)	CBD5: 3 (30)<br>CBD10: 3 (38)<br>CBD20: 0<br>Placebo: 0	CBD10: 15 (23)<br>CBD20: 15 (22)<br>Placebo: 11 (17)	N/A	CBD25: 14 (19)<br>CBD50: 12 (16)<br>Placebo: 6 (8)	CBD: 11 (13)<br>Placebo: 7 (8)	N/A
**Decreased appetite**	CBD: 2 (7)<br>Placebo: 0	CBD: 17 (28)<br>Placebo: 3 (5)	CBD10: 11 (16)<br>CBD20: 21 (26)<br>Placebo: 6 (8)	CBD5: 0<br>CBD10: 1 (13)<br>CBD20: 4 (44)<br>Placebo: 0	CBD10: 11 (17)<br>CBD20: 20 (29)<br>Placebo: 11 (17)	N/A	CBD25: 15 (20)<br>CBD50: 17 (23)<br>Placebo: 9 (12)	CBD: 11 (13)<br>Placebo: 2 (2)	N/A
**Vomiting**	N/A	CBD: 9 (15)<br>Placebo: 3 (5)	CBD10: 4 (6)<br>CBD20: 10 (12)<br>Placebo: 9 (12)	CBD5: 1 (10)<br>CBD10: 1 (13)<br>CBD20: 1 (11)<br>Placebo: 0	CBD10: 4 (6)<br>CBD20: 11 (16)<br>Placebo: 4 (6)	N/A	CBD25: 13 (17)<br>CBD50: 13 (18)<br>Placebo: 7 (9)	CBD: 9 (10)<br>Placebo: 14 (16)	CBD: 3 (18.8)<br>Placebo: 0
**Study ID**	Ben-Menachem et	Devinsky et al. 2017 (1)	Devinsky et al. 17, 2018 (2)	Devinsky et al. 14, 2018 (3)	Miller et al. 2020 (6)	O'Brien et al. 2022 (9)	Thiele et al. 2020 (8)	Thiele et al. 2018 (4)	VanLandingham et al. 2020 (7)

© 2023 Fazlollahi A et al. *JAMA Network Open.*

	al. 2020 (5)								
**Nasopharyngitis**	CBD: 2 (7)<br>Placebo: 0	N/A	CBD10: 3 (4)<br>CBD20: 9 (11)<br>Placebo: 5 (7)	CBD5: 0<br>CBD10: 1 (13)<br>CBD20: 1 (11)<br>Placebo: 1 (14)	CBD10: 4 (6)<br>CBD20: 8 (12)<br>Placebo: 5 (8)	N/A	CBD25: 11 (15)<br>CBD50: 11 (15)<br>Placebo: 12 (16)	N/A	N/A
**ALT or AST elevation**	CBD: 2 (7)<br>Placebo: 0	CBD: 12 (2)<br>Placebo: 1 (2)	CBD: 14 (9)	CBD: 6 (22)	CBD10: 3 (5)<br>CBD20: 9 (13)<br>Placebo: 0	N/A	CBD: 42 (28)<br>Placebo: 0	CBD: 20 (23)<br>Placebo: 1 (1)	CBD: 2 (13)
**γ-Glutamyltransferase elevation**	N/A	N/A	N/A	N/A	N/A	N/A	CBD25: 12 (16)<br>CBD50: 10 (14)<br>Placebo: 0	N/A	N/A
**Seizure**	N/A	N/A	N/A	N/A	N/A	N/A	CBD25: 5 (7)<br>CBD50: 8 (11)<br>Placebo: 5 (7)	N/A	N/A
**Constipation**	N/A	N/A	N/A	N/A	N/A	N/A	CBD25: 8 (11)<br>CBD50: 5 (7)<br>Placebo: 6 (8)	N/A	N/A
**Cough**	N/A	N/A	N/A	N/A	N/A	N/A	CBD25: 8 (11)<br>CBD50: 3 (4)<br>Placebo: 5 (7)	N/A	N/A
**Status epilepticus**	N/A	CBD: 3 (5)<br>Placebo: 3 (5)	CBD10: 7 (10)<br>CBD20: 4 (5)<br>Placebo: 3 (4)	N/A	CBD10: 5 (8)<br>CBD20: 7 (10)<br>Placebo: 9 (14)	N/A	N/A	CBD: 1 (1)<br>Placebo: 1 (1)	N/A
**Sedation**	N/A	N/A	N/A	CBD5: 2 (20)<br>CBD10: 0<br>CBD20: 2 (22)<br>Placebo: 0	N/A	N/A	N/A	CBD: 2 (2)	CBD: 2 (12.5)<br>Placebo: 0
**Ataxia**	N/A	N/A	N/A	CBD5: 2 (20)<br>CBD10: 0<br>CBD20: 1 (11)<br>Placebo: 0	N/A	CBD: 3 (2.4)	N/A	N/A	N/A
**Viral gastroenteritis**	N/A	N/A	N/A	CBD5: 1 (10)<br>CBD10: 0<br>CBD20: 1 (11)<br>Placebo: 1 (14)	N/A	N/A	N/A	N/A	N/A
**Study ID**	Ben-Menachem et al. 2020 (5)	Devinsky et al. 2017 (1)	Devinsky et al. 17, 2018 (2)	Devinsky et al. 14, 2018 (3)	Miller et al. 2020 (6)	O'Brien et al. 2022 (9)	Thiele et al. 2020 (8)	Thiele et al. 2018 (4)	VanLandingham et al. 2020 (7)

© 2023 Fazlollahi A et al. *JAMA Network Open.*

**Fatigue**	CBD: 3 (10)<br>Placebo: 0	CBD: 12 (20)<br>Placebo: 2 (3)	N/A	CBD5: 0<br>CBD10: 0<br>CBD20: 1 (11)<br>Placebo: 2 (29)	CBD10: 5 (8)<br>CBD20: 15 (22)<br>Placebo: 7 (11)	CBD: 7 (5.6)	N/A	N/A	N/A
**Convulsion**	N/A	CBD: 7 (11)<br>Placebo: 3 (5)	N/A	CBD5: 0<br>CBD10: 1 (13)<br>CBD20: 0<br>Placebo: 2 (29)	N/A	N/A	N/A	N/A	N/A
**Abnormal behavior**	N/A	N/A	N/A	CBD5: 3 (30)<br>CBD10: 0<br>CBD20: 0<br>Placebo: 0	N/A	N/A	N/A	N/A	N/A
**Gastroenteritis**	N/A	N/A	N/A	CBD5: 1 (10)<br>CBD10: 0<br>CBD20: 0<br>Placebo: 2 (29)	N/A	N/A	N/A	N/A	N/A
**Upper abdominal pain**	N/A	N/A	N/A	CBD5: 0<br>CBD10: 0<br>CBD20: 2 (22)<br>Placebo: 0	N/A	N/A	N/A	N/A	N/A
**Rash**	N/A	N/A	N/A	CBD5: 0<br>CBD10: 1 (13)<br>CBD20: 1 (11)<br>Placebo: 0	CBD10: 6 (9)<br>CBD20: 4 (6)<br>Placebo: 1 (2)	N/A	CBD25: 4 (5)<br>CBD50: 7 (10)<br>Placebo: 2 (3)	N/A	N/A
**Viral infection**	N/A	N/A	N/A	CBD5: 0<br>CBD10: 0<br>CBD20: 1 (11)<br>Placebo: 1 (14)	N/A	N/A	N/A	N/A	N/A
**Streptococcal Pharyngitis**	N/A	N/A	N/A	CBD5: 1 (10)<br>CBD10: 0<br>CBD20: 0<br>Placebo: 1 (14)	N/A	N/A	N/A	N/A	N/A
**Study ID**	Ben-Menachem et al. 2020 (5)	Devinsky et al. 2017 (1)	Devinsky et al. 17, 2018 (2)	Devinsky et al. 14, 2018 (3)	Miller et al. 2020 (6)	O'Brien et al. 2022 (9)	Thiele et al. 2020 (8)	Thiele et al. 2018 (4)	VanLandingham et al. 2020 (7)
**Psychomotor hyperactivity**	N/A	N/A	N/A	CBD5: 1 (10)<br>CBD10: 0<br>CBD20: 0	N/A	N/A	N/A	N/A	N/A

© 2023 Fazlollahi A et al. *JAMA Network Open.*

				Placebo: 1 (14)					
**Nausea**	CBD: 4 (14) Placebo: 0	N/A	N/A	N/A	N/A	CBD: 6 (4.8)	N/A	N/A	CBD: 3 (18.8) Placebo: 0
**Dizziness**	N/A	N/A	N/A	N/A	N/A	N/A	N/A	N/A	CBD: 2 (12.5) Placebo: 0
**Dermatitis**	N/A	N/A	N/A	N/A	N/A	N/A	N/A	N/A	CBD: 2 (12.5) Placebo: 0
**Lethargy**	N/A	CBD: 8 (13) Placebo: 3 (5)	N/A	N/A	N/A	N/A	N/A	N/A	N/A
**Aggression or Irritability**	N/A	N/A	N/A	N/A	CBD10: 3 (5) CBD20: 9 (13) Placebo: 3 (5)	N/A	N/A	N/A	N/A
**Headache**	N/A	N/A	N/A	N/A	N/A	CBD: 7 (5.6)	N/A	N/A	N/A
**Application site dryness**	N/A	N/A	N/A	N/A	N/A	CBD: 5 (4.0)	N/A	N/A	N/A
**Urinary tract infection**	N/A	N/A	N/A	N/A	N/A	CBD: 4 (3.2)	N/A	N/A	N/A
**Anxiety**	N/A	N/A	N/A	N/A	N/A	CBD: 4 (3.2)	N/A	N/A	N/A
**Application site pruritus**	N/A	N/A	N/A	N/A	N/A	CBD: 3 (2.4)	N/A	N/A	N/A
**Application site moderate erythema**	N/A	N/A	N/A	N/A	N/A	CBD: 3 (2.4)	N/A	N/A	N/A
**Oropharyngeal pain**	N/A	N/A	N/A	N/A	N/A	CBD: 3 (2.4)	N/A	N/A	N/A
**Thermal burns**	N/A	N/A	N/A	N/A	N/A	CBD: 3 (2.4)	N/A	N/A	N/A

Abbreviations: ALT: alanine aminotransferase; AST: aspartate aminotransferase; CBD: cannabidiol, N/A: not applicable.

© 2023 Fazlollahi A et al. *JAMA Network Open.*

eTable 6. Graded adverse events in the meta-analysis.

Classification*	Adverse event	Mild		Moderate		Severe	
		RR (95% CI)	P-value	RR (95% CI)	P-value	RR (95% CI)	P-value
Infections and infestations	Nasopharyngitis	1.025 (0.634, 1.658)	0.918	N/A	N/A	N/A	N/A
Gastrointestinal disorders	Diarrhea	1.707 (1.206, 2.416)	0.003	1.855 (0.721, 4.773)	0.200	1.894 (0.301, 11.930)	0.496
	Vomiting	0.887 (0.545, 1.445)	0.631	1.480 (0.576, 3.803)	0.415	N/A	N/A
Nervous system disorders	Somnolence	1.653 (0.891, 3.067)***	0.111	3.620 (1.449, 9.044)	0.006	1.828 (0.300, 11.138)	0.513
General disorders	Pyrexia	1.194 (0.783, 1.820)	0.409	1.121 (0.844, 1.490)	0.431	N/A	N/A
Metabolism and nutrition disorders	Decreased appetite	1.503 (0.970, 2.329)	0.068	3.252 (1.197, 8.832)	0.021	1.799 (0.365, 8.855)	0.470

Abbreviations: RR: risk ratio; CI: confidence interval; N/A: not applicable

* We categorized the adverse events according to the Common Terminology Criteria for Adverse Events (CTCAE), Version 5.0 (10).

** We included adverse events that were reported in three or more of the included studies.

*** The values of the random-effects model are reported.

© 2023 Fazlollahi A et al. *JAMA Network Open.*

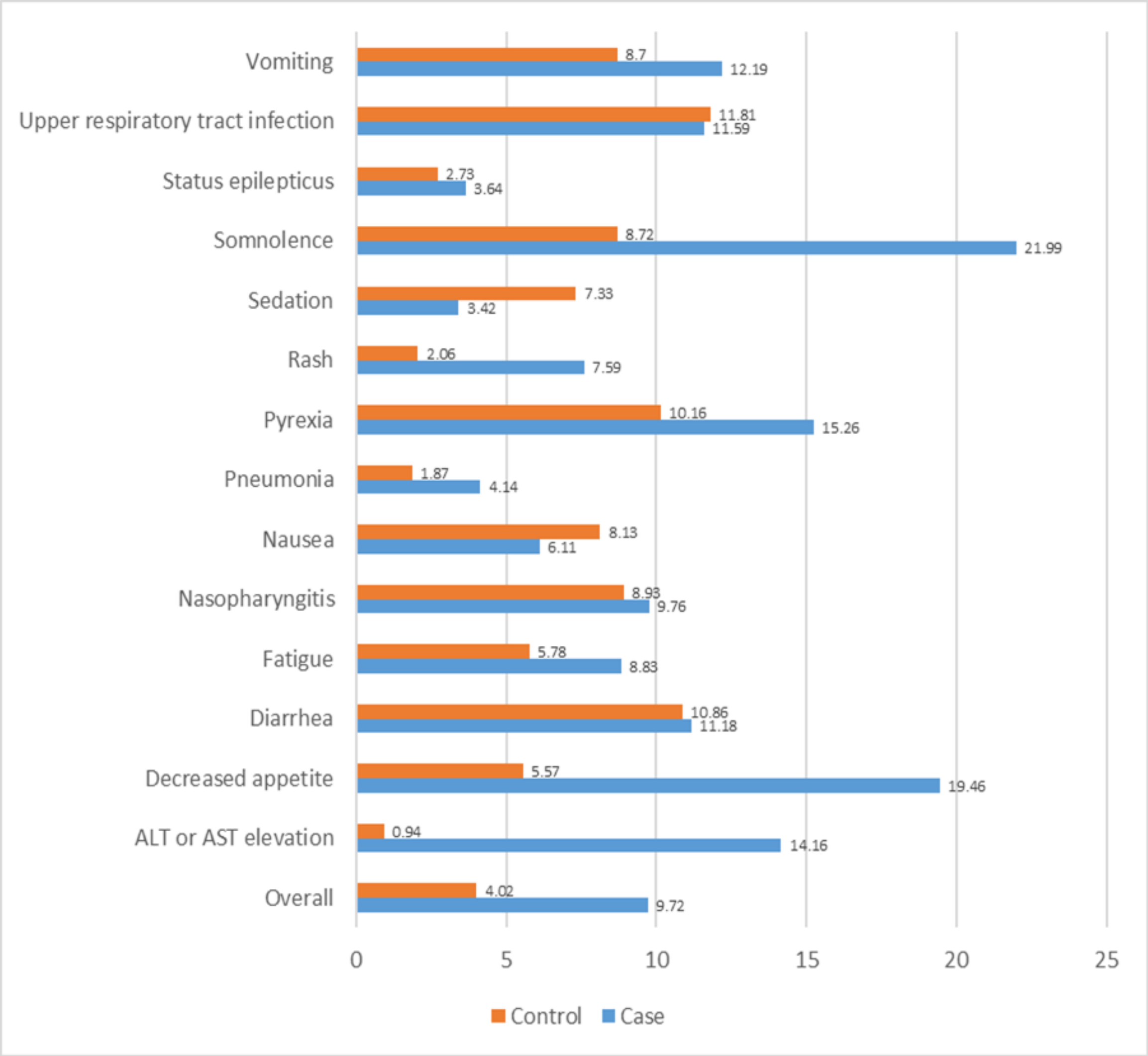

eFigure 1. Percentages of any-grade adverse events for the cannabidiol and control groups.

© 2023 Fazlollahi A et al. *JAMA Network Open.*

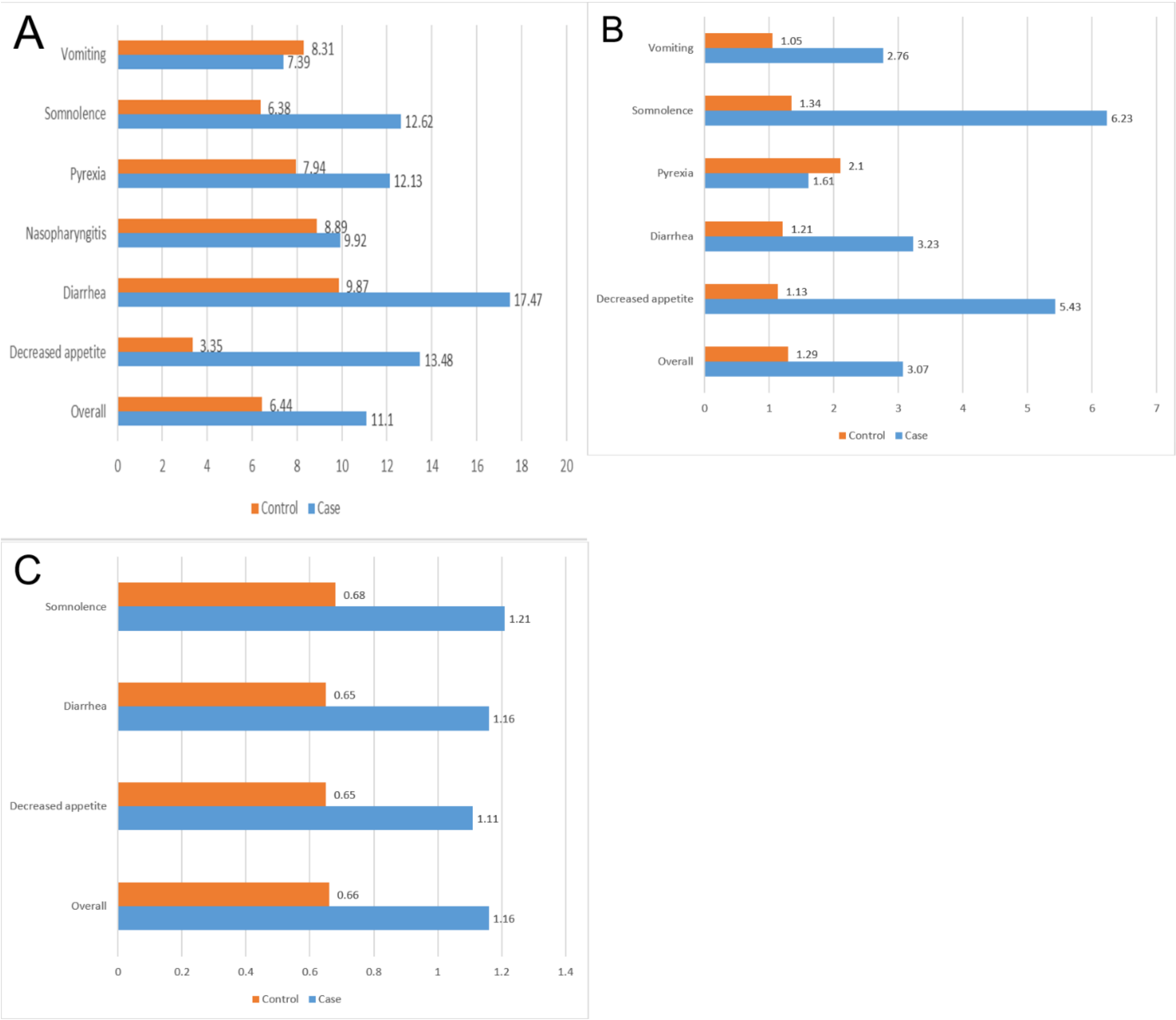

eFigure 2. Percentages of mild (A), moderate (B) and severe (C) adverse events for the cannabidiol and control groups.

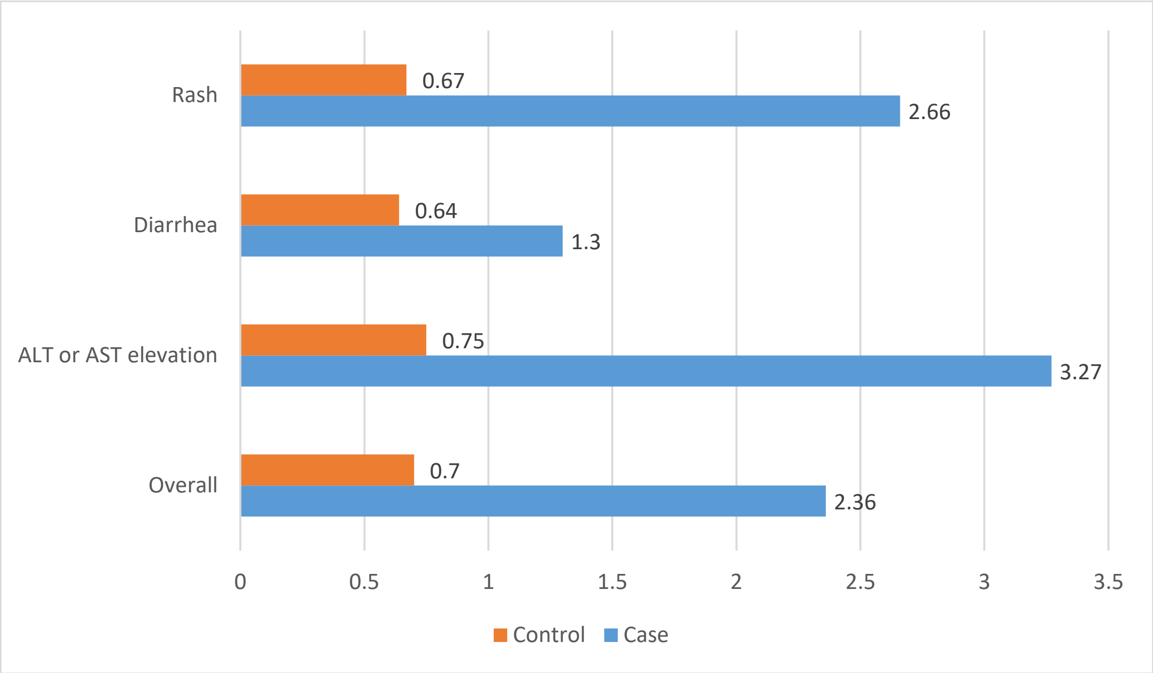

eFigure 3. Percentages of adverse events leading to the discontinuation the trial for the cannabidiol and control groups.

© 2023 Fazlollahi A et al. *JAMA Network Open.*

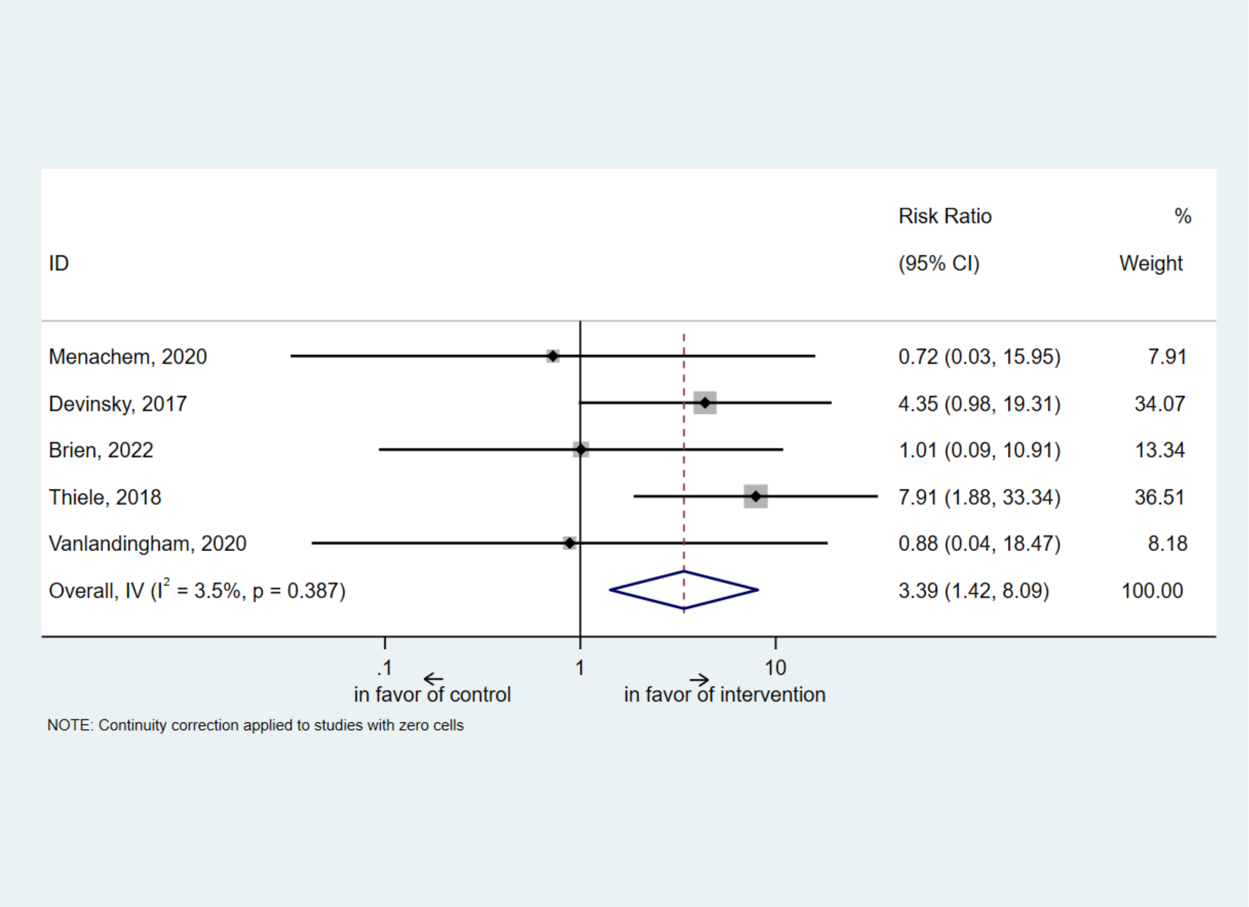

eFigure 4. Forest plot of the risk ratios for severe grade adverse events for the cannabidiol and control groups. CI: confidence interval; IV: inverse variance. Brien, 2022 has a high risk of bias; Devinsky, 2017; Menachem, 2020 and Vanlandingham, 2020 have some concerns; and Thiele, 2018 has a low risk of bias.

© 2023 Fazlollahi A et al. *JAMA Network Open.*

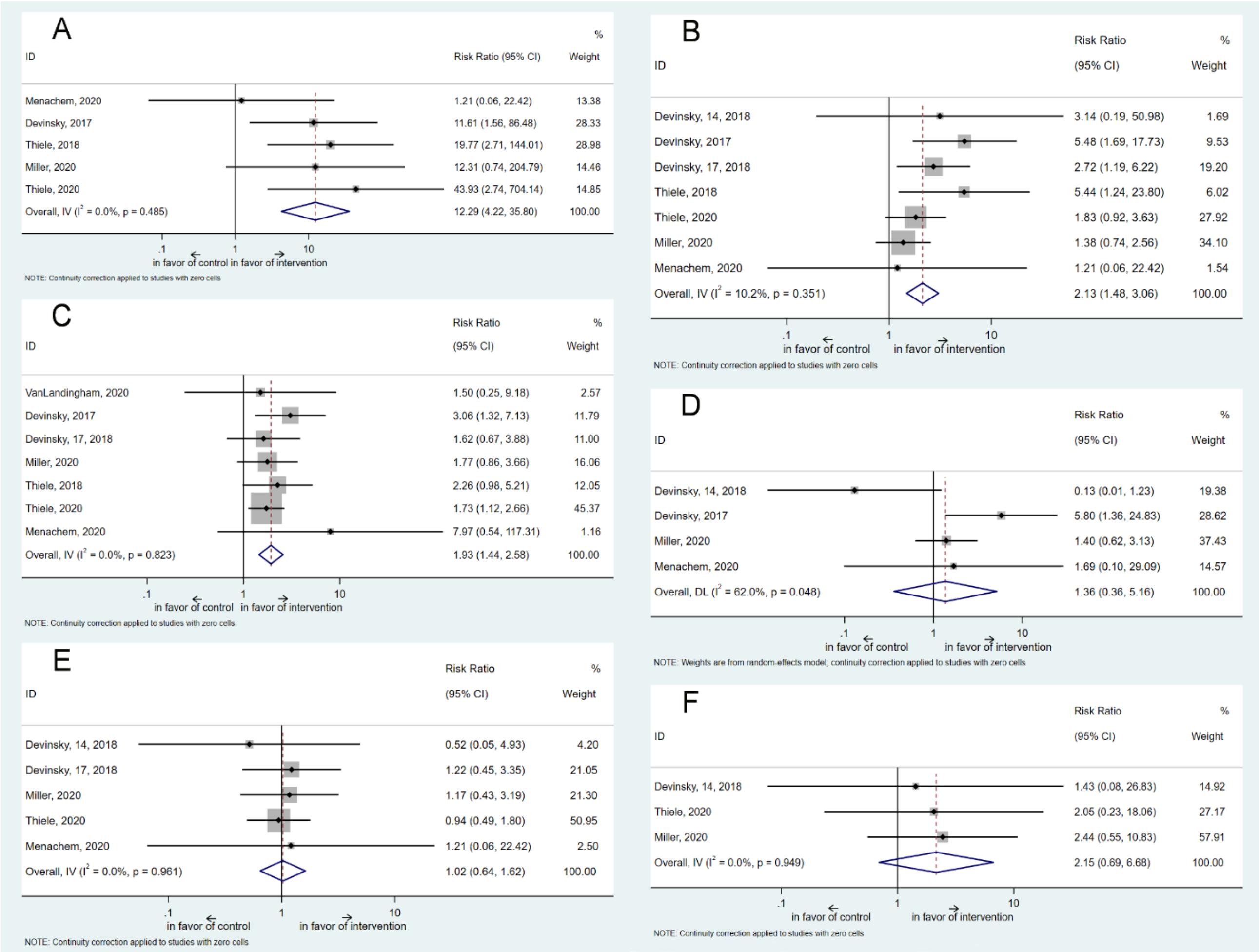

eFigure 5. Forest plots of the risk ratios for any-grade adverse events, including ALT or AST elevation (A), decreased appetite (B), diarrhea (C), fatigue (D), nasopharyngitis (E), and pneumonia (F) for the cannabidiol and control groups. CI: confidence interval; IV: inverse variance; DL: DerSimonian and Laird. Devinsky, 17, 2018 and Devinsky, 14, 2018 have a high risk of bias; Devinsky, 2017; Menachem, 2020 and Vanlandingham, 2020 have some concerns; and Thiele, 2018; Miller, 2020 and Thiele, 2020 have a low risk of bias.

© 2023 Fazlollahi A et al. *JAMA Network Open.*

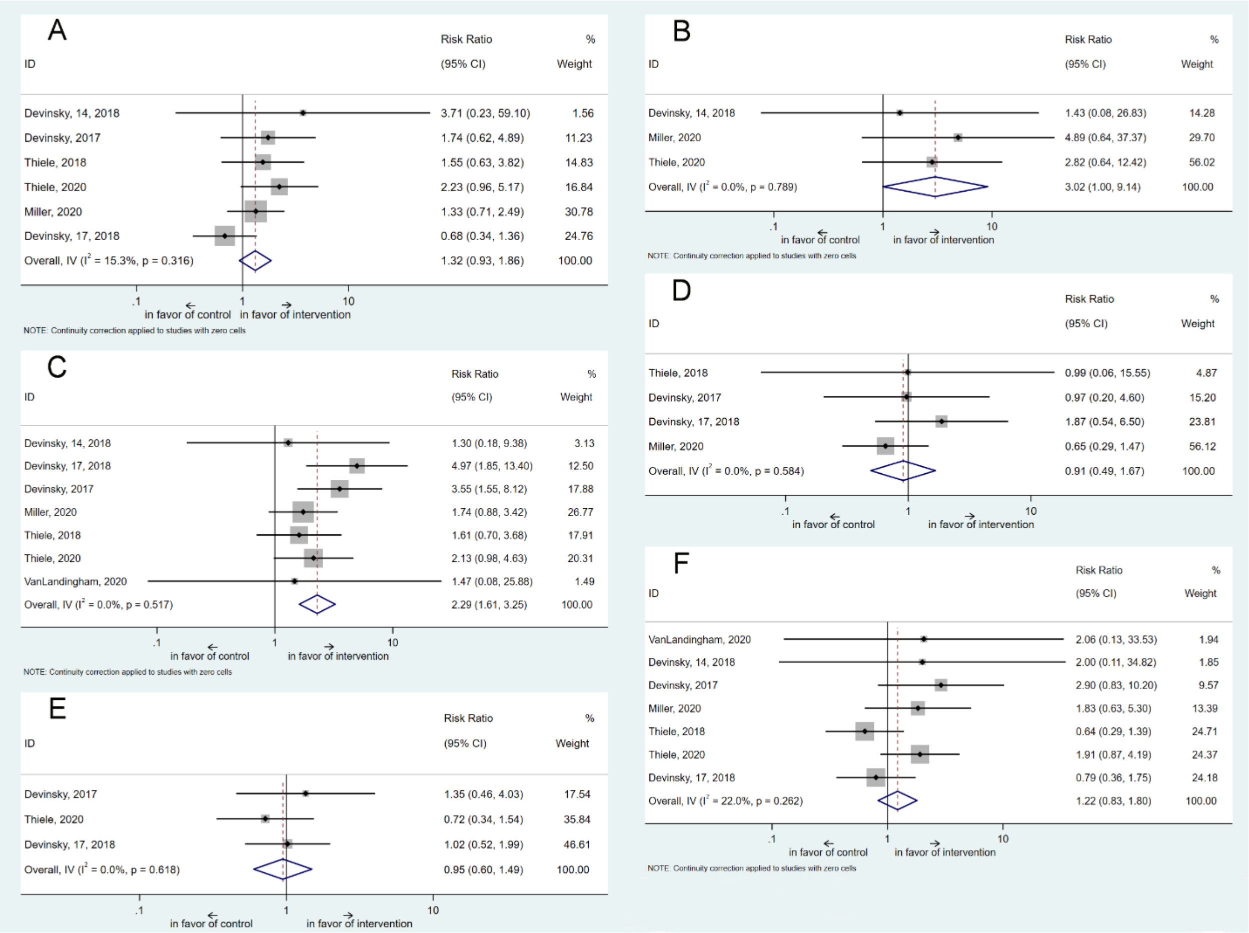

eFigure 6. Forest plots of the risk ratios for any-grade adverse events, including pyrexia (A), rash(B), somnolence (C), status epilepticus (D), upper respiratory tract infection (E), vomiting (F), for the cannabidiol and control groups. CI: confidence interval; IV: inverse variance. Devinsky, 17, 2018 and Devinsky, 14, 2018 have a high risk of bias; Devinsky, 2017 and Vanlandingham, 2020 have some concerns; and Thiele, 2018; Miller, 2020 and Thiele, 2020 have a low risk of bias.

© 2023 Fazlollahi A et al. *JAMA Network Open.*

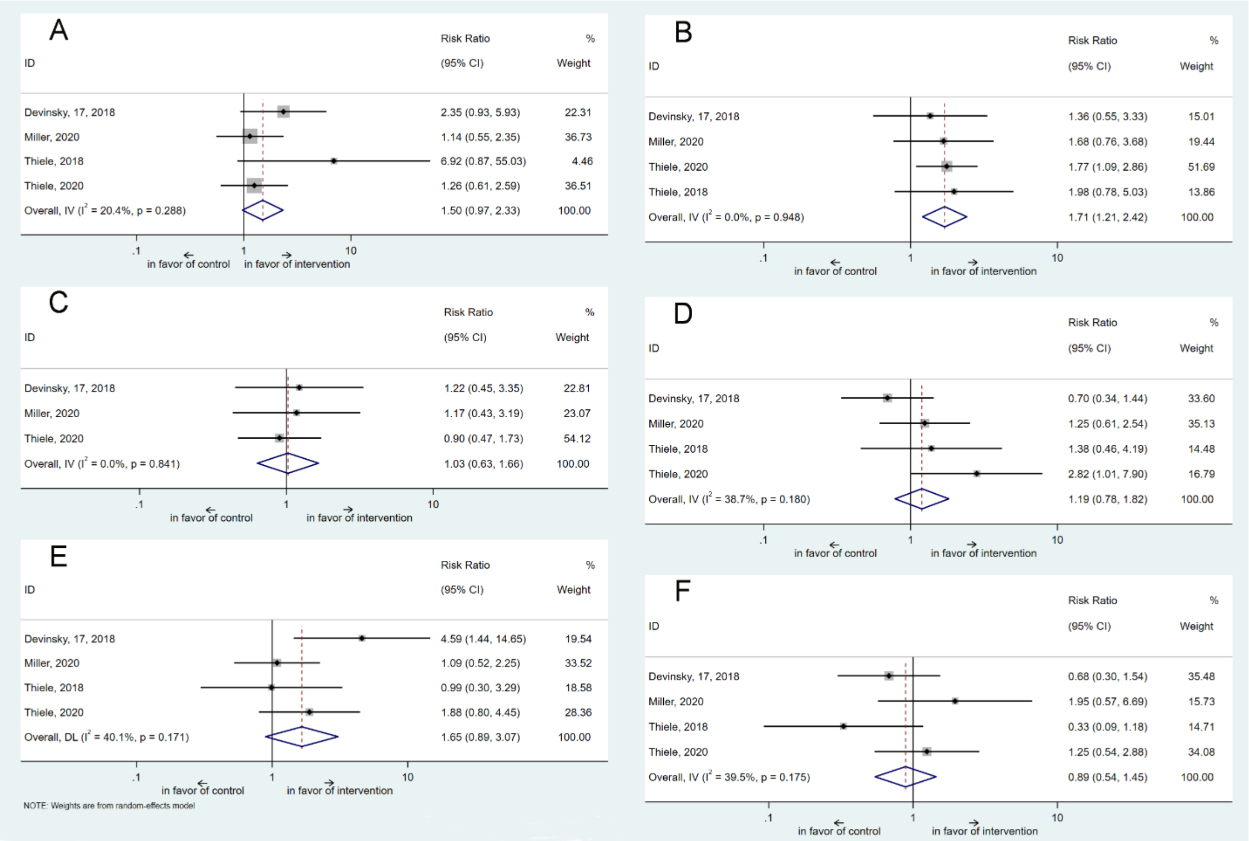

eFigure 7. Forest plot of the risk ratios for mild adverse events, including decreased appetite (A), diarrhea (B), nasopharyngitis (C), pyrexia (D), somnolence (E), and vomiting (F) for the cannabidiol and control groups. CI: confidence interval; IV: inverse variance, DL: DerSimonian and Laird. Devinsky, 17, 2018 has a high risk of bias; and Thiele, 2018; Miller, 2020 and Thiele, 2020 have a low risk of bias.

© 2023 Fazlollahi A et al. *JAMA Network Open.*

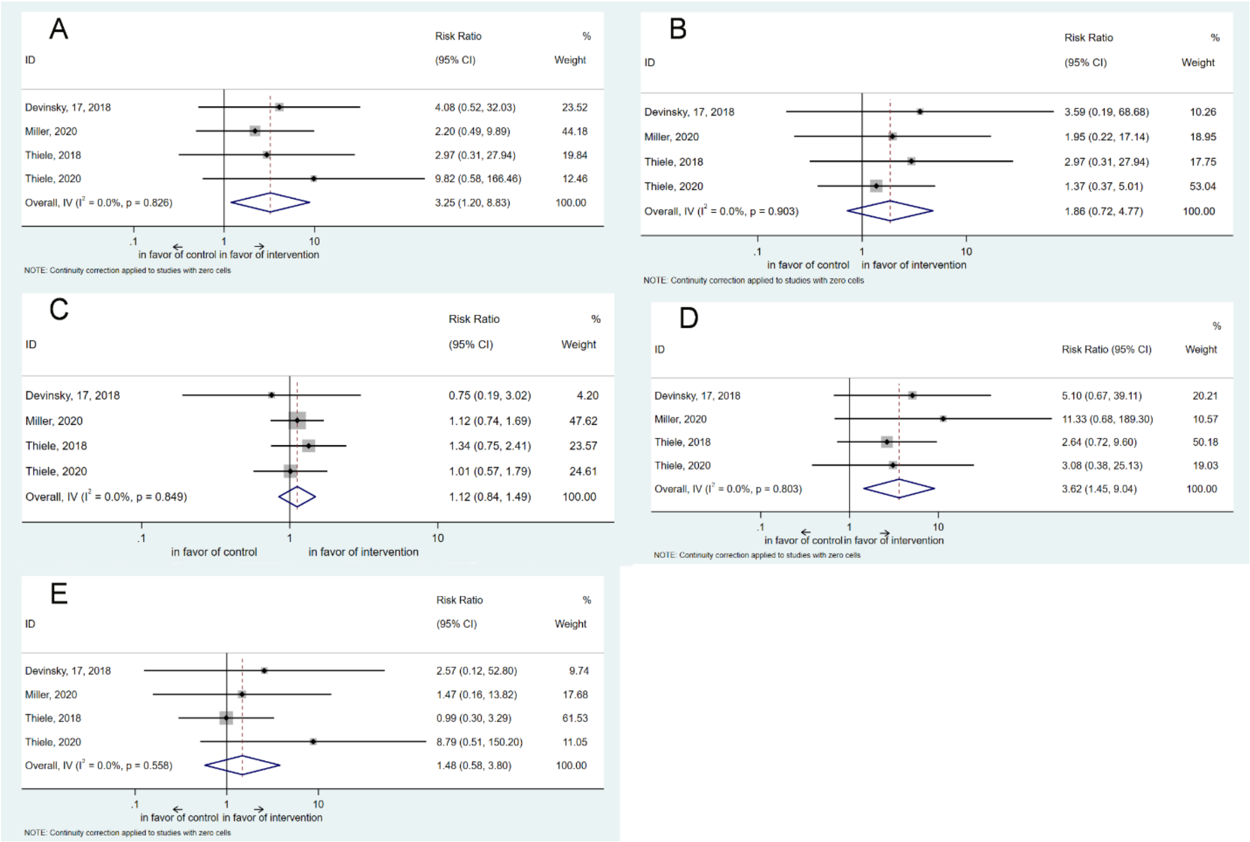

eFigure 8. Forest plot of the risk ratios for moderate adverse events, including decreased appetite (A), diarrhea (B), pyrexia (C), somnolence (D), and vomiting (E) for the cannabidiol and control groups. CI: confidence interval; IV: inverse variance. Devinsky, 17, 2018 has a high risk of bias; and Thiele, 2018; Miller, 2020 and Thiele, 2020 have a low risk of bias.

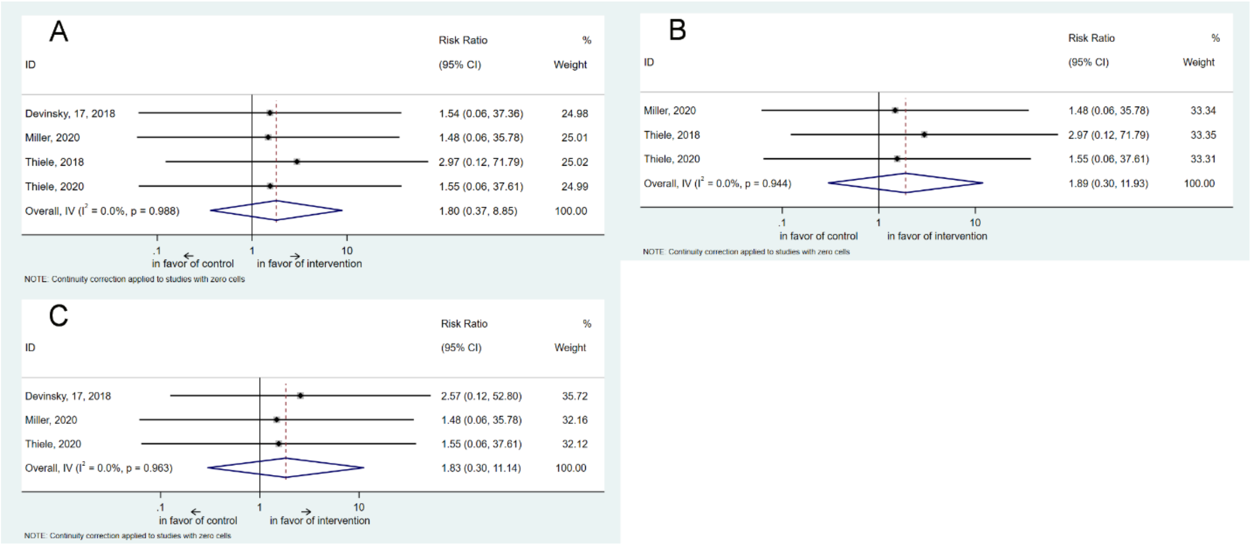

eFigure 9. Forest plot of the risk ratios for severe adverse events, including decreased appetite (A), diarrhea (B), somnolence (C), for the cannabidiol and control groups. CI: confidence interval; IV: inverse variance. Devinsky, 17, 2018 has a high risk of bias; and Thiele, 2018; Miller, 2020 and Thiele, 2020 have a low risk of bias.

© 2023 Fazlollahi A et al. *JAMA Network Open.*

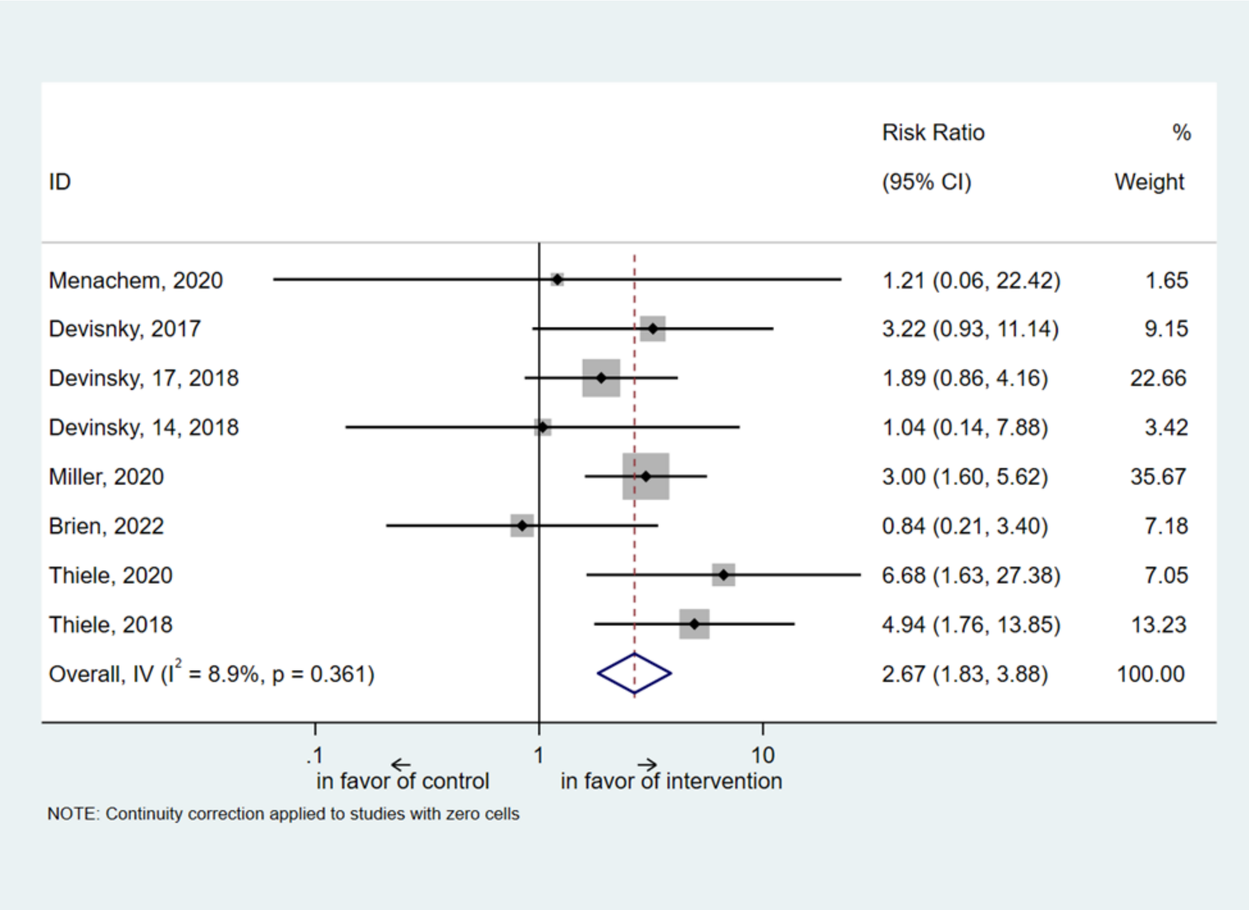

eFigure 10. Forest plot of the risk ratio for serious adverse events for the cannabidiol and control groups. CI: confidence interval; IV: inverse variance. Devinsky, 17, 2018; Devinsky, 14, 2018 and Brien, 2022 have a high risk of bias; Devinsky, 2017 and Menachem, 2020 have some concerns; and Thiele, 2018; Miller, 2020 and Thiele, 2020 have a low risk of bias.

© 2023 Fazlollahi A et al. *JAMA Network Open.*

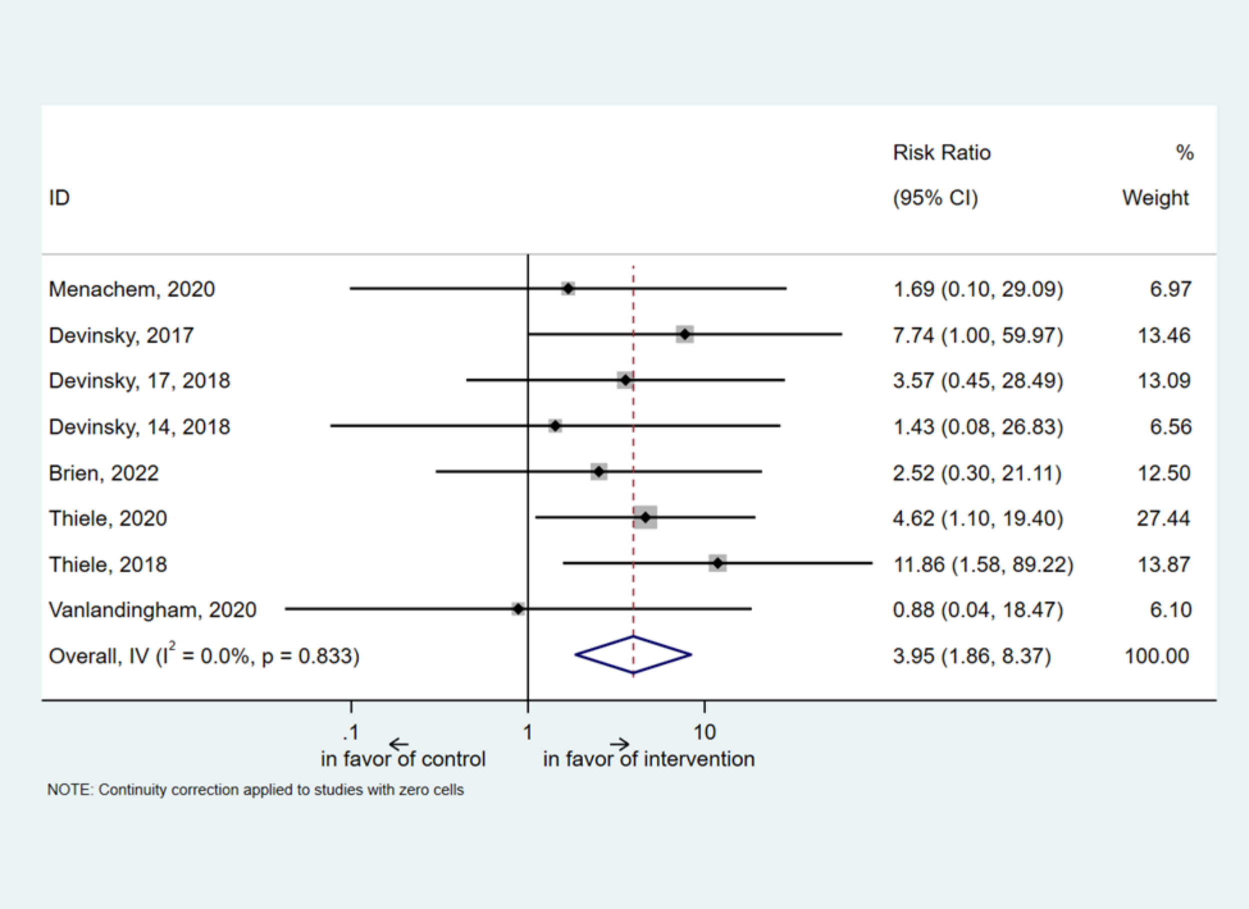

eFigure 11. Forest plot of the risk ratio for adverse events leading to the discontinuation of the trial for the cannabidiol and control groups. CI: confidence interval; IV: inverse variance. Devinsky, 17, 2018; Devinsky, 14, 2018 and Brien, 2022 have a high risk of bias; Devinsky, 2017; Menachem, 2020 and Vanlandingham, 2020 have some concerns; and Thiele, 2018 and Thiele, 2020 have a low risk of bias.

© 2023 Fazlollahi A et al. *JAMA Network Open.*

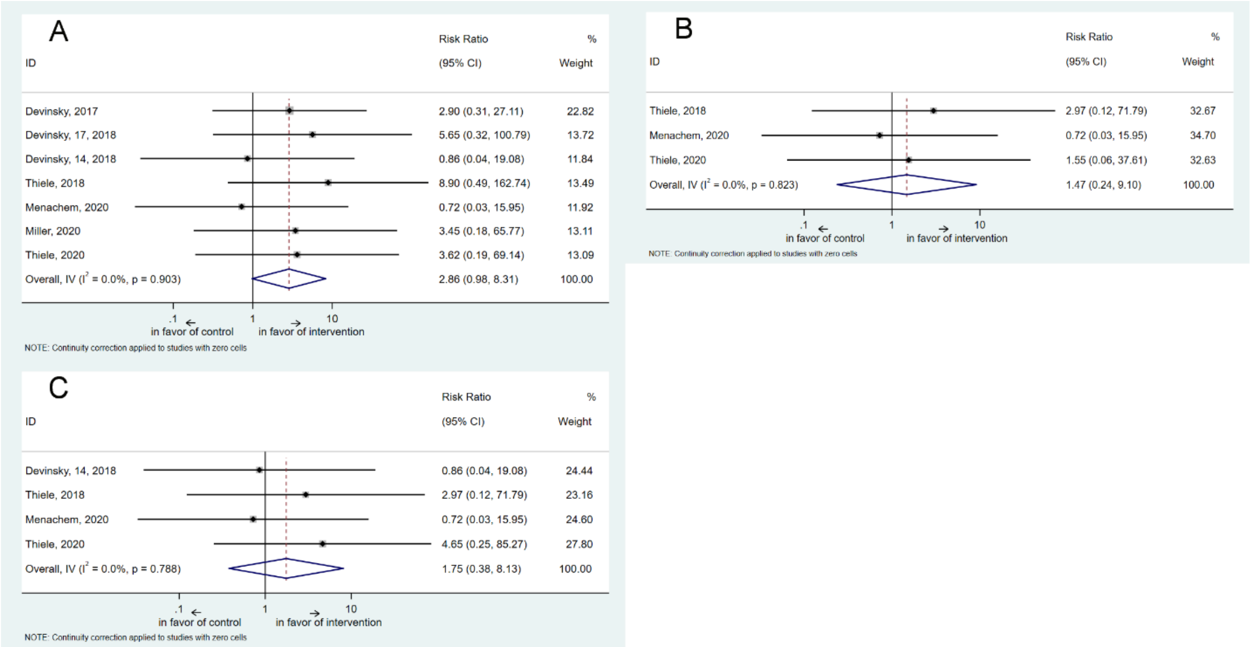

eFigure 12. Forest plot of the risk ratios for adverse events leading to the discontinuation of the trial, including ALT or AST elevation (A), diarrhea (B), and rash (C) for the cannabidiol and control groups. CI: confidence interval; IV: inverse variance. Devinsky, 17, 2018 and Devinsky, 14, 2018 have a high risk of bias; Devinsky, 2017 and Menachem, 2020 have some concerns; and Thiele, 2018; Miller, 2020 and Thiele, 2020 have a low risk of bias.

© 2023 Fazlollahi A et al. *JAMA Network Open.*

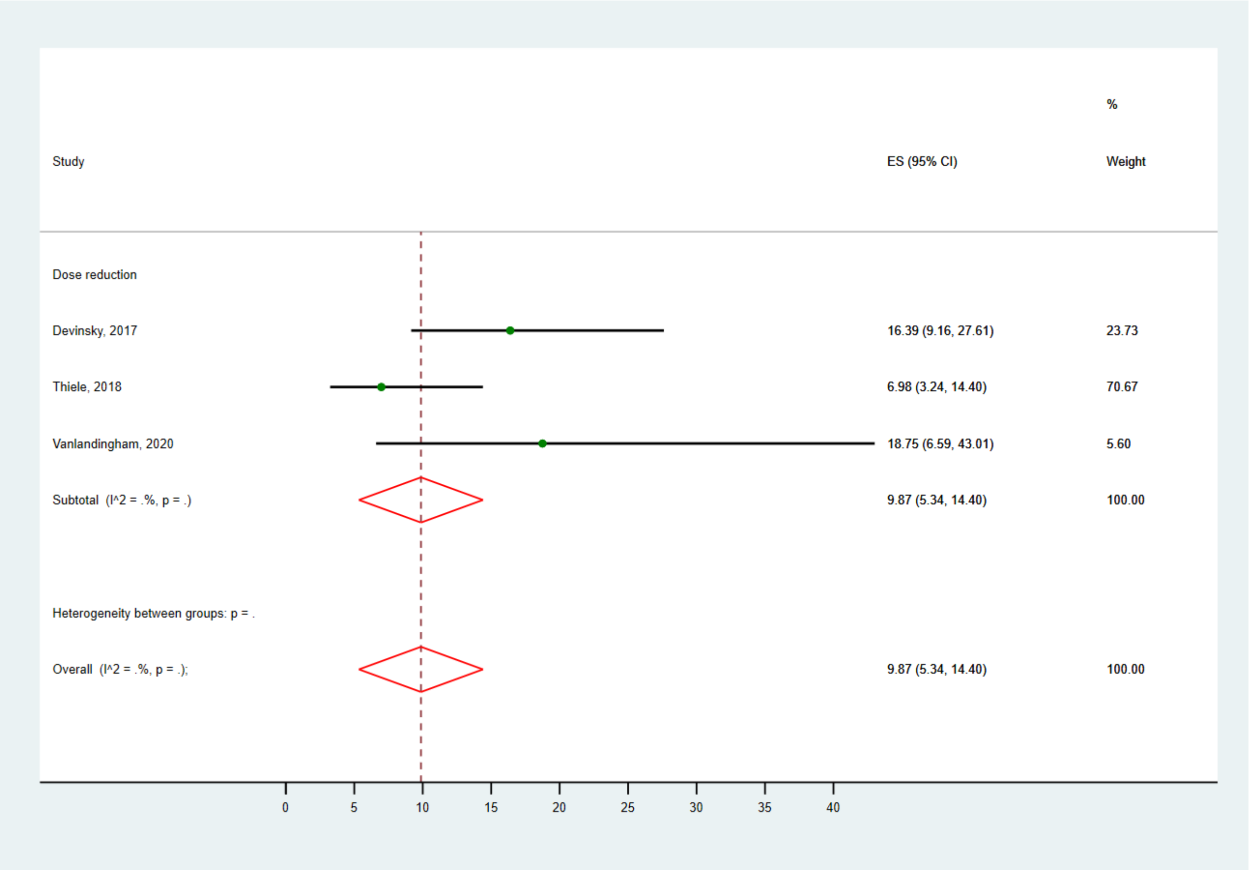

eFigure 13. Forest plot of the risk ratios for adverse events leading to dose reduction for the cannabidiol and control groups. CI: confidence interval; IV: inverse variance. Devinsky, 2017 and Vanlandingham, 2020 have some concerns; and Thiele, 2018 has a low risk of bias.

© 2023 Fazlollahi A et al. *JAMA Network Open.*

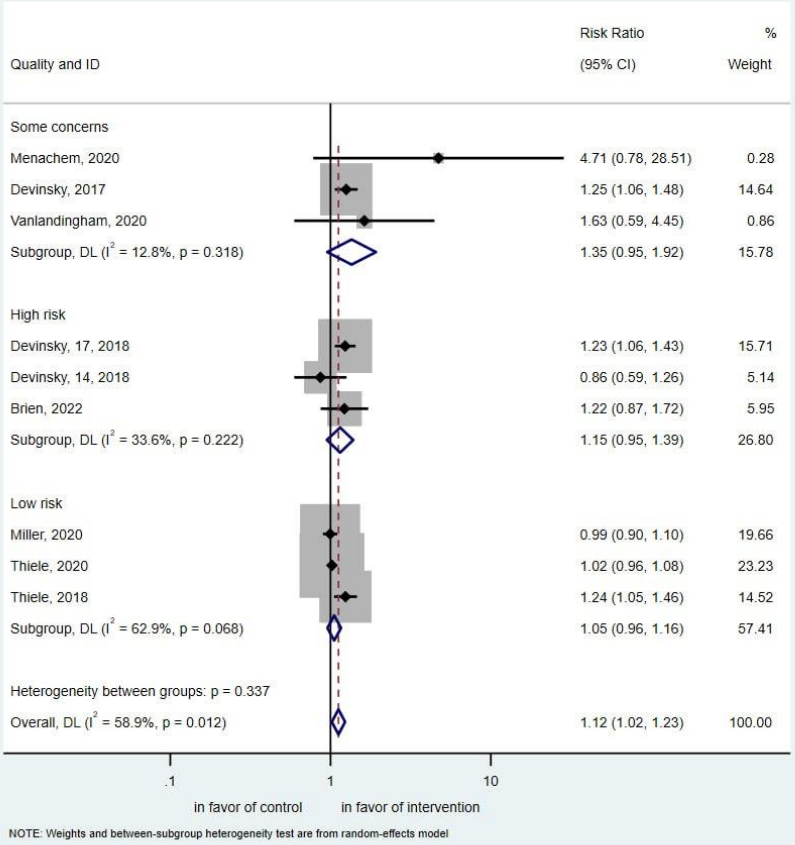

eFigure 14. Forest plot of the risk ratio for any-grade adverse events for the cannabidiol and control groups by quality of the included studies. DL: DerSimonian and Laird; CI: confidence interval.

© 2023 Fazlollahi A et al. *JAMA Network Open.*

e**References**

1. Devinsky O, Cross JH, Laux L, Marsh E, Miller I, Nabbout R, et al. Trial of Cannabidiol for Drug-Resistant Seizures in the Dravet Syndrome. N Engl J Med. 2017;376(21):2011-20.
2. Devinsky O, Patel AD, Cross JH, Villanueva V, Wirrell EC, Privitera M, et al. Effect of Cannabidiol on Drop Seizures in the Lennox-Gastaut Syndrome. N Engl J Med. 2018;378(20):1888-97.
3. Devinsky O, Patel AD, Thiele EA, Wong MH, Appleton R, Harden CL, et al. Randomized, dose-ranging safety trial of cannabidiol in Dravet syndrome. Neurology. 2018;90(14):E1204-+.
4. Thiele EA, Marsh ED, French JA, Mazurkiewicz-Beldzinska M, Benbadis SR, Joshi C, et al. Cannabidiol in patients with seizures associated with Lennox-Gastaut syndrome (GWPCARE4): a randomised, double-blind, placebo-controlled phase 3 trial. Lancet. 2018;391(10125):1085-96.
5. Ben-Menachem E, Gunning B, Cabrera CMA, VanLandingham K, Crockett J, Critchley D, et al. A Phase II Randomized Trial to Explore the Potential for Pharmacokinetic Drug-Drug Interactions with Stiripentol or Valproate when Combined with Cannabidiol in Patients with Epilepsy. Cns Drugs. 2020;34(6):661-72.
6. Miller I, Scheffer IE, Gunning B, Sanchez-Carpintero R, Gil-Nagel A, Perry MS, et al. Dose-Ranging Effect of Adjunctive Oral Cannabidiol vs Placebo on Convulsive Seizure Frequency in Dravet Syndrome A Randomized Clinical Trial. JAMA Neurol. 2020;77(5):613-21.
7. Vanlandingham K, Crockett J, Taylor L, Morrison G, Maguire M. A Phase 2, Double-Blind, Placebo-Controlled Trial to Investigate Potential Drug-Drug Interactions Between Cannabidiol and Clobazam. The Journal of Clinical Pharmacology. 2020;60:1-10.
8. Thiele EA, Bebin EM, Bhathal H, Jansen FE, Kotulska K, Lawson JA, et al. Add-On Cannabidiol Treatment for Drug-Resistant Seizures in Tuberous Sclerosis Complex A Placebo-Controlled Randomized Clinical Trial. JAMA Neurol. 2021;78(3):285-92.
9. O'Brien TJ, Berkovic SF, French JA, Messenheimer JA, Sebree TB, Bonn-Miller MO, et al. Adjunctive Transdermal Cannabidiol for Adults With Focal Epilepsy A Randomized Clinical Trial. JAMA Netw Open. 2022;5(7):12.
10. Health UDo, Services H. Common Terminology Criteria for Adverse Events. Version 5.0. Published November 27, 2017. 2020.

© 2023 Fazlollahi A et al. *JAMA Network Open.*
