# Supplementary material for: Adverse Events of Cannabidiol Use in Patients With Epilepsy: A Systematic Review and Meta-analysis
Source: JAMA Netw Open. 2023 Apr 20;6(4):e239126. doi: 10.1001/jamanetworkopen.2023.9126 (PMC10119734; doi:10.1001/jamanetworkopen.2023.9126)
Supplement: Supplement 2. — Data Sharing Statement [file jamanetwopen-e239126-s002.pdf]

# Data Sharing Statement

Fazlollahi. Adverse Events of Cannabidiol Use in Patients With Epilepsy. *JAMA Netw Open*. Published April 20, 2023. doi:10.1001/jamanetworkopen.2023.9126

**Data**
**Data available:** No

**Additional Information**
**Explanation for why data not available:** The data that support the findings of this study are available from the corresponding author upon reasonable request.
